# Influence of Weather Conditions in the Northwestern Russian Federation on Flax Fiber Characters According to the Results of a 30-Year Study

**DOI:** 10.3390/plants13060762

**Published:** 2024-03-07

**Authors:** Andrey V. Pavlov, Elizaveta A. Porokhovinova, Anastasia A. Slobodkina, Inna I. Matvienko, Natalya V. Kishlyan, Nina B. Brutch

**Affiliations:** 1 Department of Oil and Fiber Crops, N.I. Vavilov All-Russian Institute of Plant Genetic Resources (VIR), St. Petersburg 19000, Russia; avpavlov77@yandex.ru (A.V.P.); n.brutch@vir.nw.ru (N.B.B.); 2 Department of Genetics, N.I. Vavilov All-Russian Institute of Plant Genetic Resources (VIR), St. Petersburg 19000, Russia

**Keywords:** VIR flax collection, fiber flax, seed productivity, flax fiber, prediction of fiber yield and quality

## Abstract

Weather has significant impact on plant growth and development. It is important to analyze the influence of changing climate conditions on the expression of plant agronomic characters. Two flax varieties were grown from 1987 to 2018 in the Northwest of Russia. Weather conditions and their influence on flax agronomic characters were analyzed using the variance and correlations analyses. Significant influence of conditions of a particular year on the manifestation of all evaluated characters was revealed. Starting from June, high temperatures accelerate plant development at all stages. Prolongation of the germination-flowering period is most important for improving fiber productivity, while fast ripening in hot weather after flowering is preferable for the formation of high-quality fiber. Such data give a possibility to predict the yield amount and quality. The use of weather conditions data also makes possible a comparison of the results obtained in different years. The suggested method of classifying meteorological conditions of a year can be used in other genebanks for systematizing and analyzing the results of crop evaluation in the field. The correlation analysis revealed 3 correlated pleiades, namely (1) of productivity, (2) of fiber quality and yield, and (3) of the growing season phase durations, the sums of active temperatures and precipitation during each period. The great influence of growing conditions on the economically valuable traits indicates the necessity of searching for genotypes with stable character manifestations for breeding new varieties with stable yields and good fiber quality.

## 1. Introduction

Weather conditions have a great impact on the development and yield of flax. Very favorable conditions for its cultivation have historically developed in the Non-Chernozem zone of Russia. For many centuries, flax farming provided up to 70% of the total income from crop production, occupying only 6–8% of sown areas [1]. Recently, significant weather anomalies have been observed more and more often. So, cultivation of varieties capable of adapting to rapid changes in weather conditions continues to be the most effective way of increasing the crop yield and quality of the obtained products. However, the industrially cultivated modern flax varieties realize only 30–35% of their inherent yield potential. This is mainly due to the significant influence of adverse weather factors [2], so the creation and cultivation of highly plastic varieties would substantially neutralize the degree of negative impact of fluctuating weather conditions. A successful solution of this problem should be based on understanding the influence of environmental conditions on the plant growth and development. Though molecular mechanisms of plant responses to different abiotic stresses are now intensively evaluated [3], the complex influence of weather conditions still needs to be clarified. For successful crop production, it is very important to understand the environment requirements of the crop in different stages of development and, on the other hand, outline the main features that are required for the newly developed varieties to withstand unfavorable and changing weather conditions.

The life of each plant begins with germination. The optimum temperature for fiber flax germination is +10–13 °C [4], and +20–25 °C for linseed [5]. Temperature of +45 °C completely suppresses the embryo development [4]. Cold delays the emergence of seedlings, but does not affect field germination ability [6]. Laboratory experiments at +1–4 °C delayed full germination for 7–8 days in fiber flax and for 5–6 days in the linseed [7]. Successful germination needs sufficient moisture in the soil, necessary for the rapid hydrolysis of storage proteins and the conversion of fats into sugars [8]. Soil moisture content of 40–60% is optimal for flax sowing [4].

Plant germination is associated with the phase of vernalization. Though *L. usitatissimum* does not have true winter forms [9], many scientists distinguish a group of semi-winter flaxes of southern origin that require prolonged cooling and are cultivated as a winter crop. In this case, a delay of development at low temperatures helps to avoid damage by freezing [10].

Young flax plants withstand short-term frosts of up to −5 °C [11]. But it causes a lag in growth, a delay of flowering, the development of a greater plant height, a decrease in fiber quality [11], and delay of maturation [12]. The next phenological stage, specific for flax, is the herringbone stage [8]. It is characterized by intensive development of the roots and slow stem growth and a reduced metabolic rate [11]. This period coincides with the so-called light stage, during which flax plants express sensitivity to the photoperiod. Cultivated flax is a long-day plant [13], but numerous differences in the degree of response to photoperiod reduction were found among fiber flax and linseed [14,15].

At the end of the light stage and the appearance of flower tubercles, the formation of leaf primordia stops. The phase of rapid intercalary growth of the stem and branches of the future inflorescence begins [8]. In parallel, the formation of bast fiber bundles occurs [8]. Warm and dry weather conditions, prevailing from late spring onwards, caused excessive shortening of the growing cycle of spring crops, with negative consequences for seed and fiber yield and quality [16]. The May–June period was found to be critical for the linseed development because of the accelerated plant growth both in terms of height and branching; precipitation during this period is a key factor for obtaining good yields [17].

The period from the beginning of the generative phase to the end of flowering is especially important in the ontogenesis of flax, since this is the time of not only a rapid increase in the size of vegetative organs, but also of the most intensive accumulation of biomass and of determination of the inflorescence size and the number of flowers in it [11]. A lack of moisture in this period significantly reduces the number of fibrous bundles in the stem, and thus the yield and fiber quality decrease [11]. High temperatures during flowering reduce the number of bolls [18], the seed yield, oil content and quality [19].

Maturation begins after fertilization and initiation of embryo development. Low temperatures during this period slow down vegetation and contribute to the development of diseases. In the European part of Russia, the main climatic factor accelerating flax vegetation is an increase in air temperature, namely the temperatures above +15 °C [20]. During the fiber maturation, temperature of +18–20 °C is the most favorable [11]. Flax yield is positively affected by a longer vegetative period. A sufficient amount of water is most important during the budding stage, followed by the flowering and fruiting stages [21,22]. Humidity is of particular importance for flax growth. Droughts cause the termination of many physiological processes, growth inhibition, and a decrease in yield and its quality [23,24].

Though the influence of individual environmental factors on flax growth and development has been evaluated well, the complex genotype–environment interaction has not been studied sufficiently. Different experiments showed different results. The analysis of variance carried out for many characters, including the number of days before maturation and plant height, showed a high significance of the interaction between the genotype and the place of cultivation [25]. The weather conditions in different years had a significant influence on the fiber quality characters [26] and plant height [27]. The combined effect of various factors on flax yield was demonstrated in field conditions. The influence of the variety genotype on the formation of yield increases significantly under favorable weather conditions, while under unfavorable hydrothermal parameters, the influence of potassium content in the soil and its acidity increases [28]. Our previous experiments also showed diversity in the stability of genotype character expression in different environmental conditions. So, the results of analyses of the genotypes, growing conditions and their interactions influence the agronomic characters of both fiber flax and linseed depend on the characters of the evaluated genotypes and (or) variability of growing conditions [29,30]. The duration of the main periods of vegetation most often depends primarily on weather conditions. The height of plants is 55–80% determined by the genotype. The fiber yield and 1000 seed weight are usually determined by the genotype. Characters of fiber quality in different experiments showed significantly different levels of the influence of genotypes, conditions of the year and their interaction.

An analysis of the manifestation of economically valuable traits is also of great importance for breeding. The interdependency of characters is a condition for the reliability of existence of an organism and arises on the basis of selection of the most stable individuals, but it is not absolute, since the independence of some processes from each other is equally necessary for the organism [31]. Correlations between characters are distinguished by the types of their relationships. Phenotypic correlations reflect the relationship of characters in a population where variation is a consequence of both genetic and environmental differences. Ecological correlations calculated on the basis of the expression of plant characters of one genotype in specific environmental conditions demonstrate a component of trait covariance that depends only on the heterogeneity of growing conditions. A genotypic correlation, on the contrary, is a covariance based solely on genetic differences. It can be calculated as a correlation between the average values of genotypes’ characters [32]. Strong genotypic correlations indicate the potential for combining desirable traits in one variety, as well as selecting breeding material by estimating only some easily identifiable traits. Strong ecological correlations show the reliability of changes in various characters under the influence of environmental variations.

The information available in the literature about the correlations between flax characters is sketchy and does not give a complete picture of their interdependency because often the authors do not separate the genotypic and environmental components of the links. When testing correlations between flax characters, the main attention is paid to the assessment of genotypic relationships. First of all, great importance is attached to establishing the possibility of selecting early ripening plants based on early flowering. However, various studies have obtained diametrically opposed results of assessing correlations between the duration of the two main periods of flax development. Apparently, these differences are related to the use of very diverse groups of the analyzed accessions.

One of the main components of fiber flax productivity is the plant height. Numerous experiments have shown that in different accessions, its relationship with other characters varies greatly, but in all cases, strong positive correlations were observed between the total height and length of the stem, height and weight of the stem [33]. Similar results were obtained in our previous experiments [29].

Evaluation of the correlation between earliness and height of flax plants has been conducted for a long time. Researchers have approached this problem from different perspectives. Having collected and evaluated about 1.5 thousand flax genotypes from various regions of the world, N.I. Vavilov [34] found that, in general, cultivated flax of the species *L. usitatissimum* has a negative correlation between the growing season duration and plant height. The shorter is the growing season, the taller are the flax plants, the closer they are to the normal type of fiber flax, the less is their branching, and the less is the production of bolls and seeds. With the lengthening of the growing season, the number of inflorescence branches, the number of stems, and the number of bolls increase, while the plant height decreases accordingly. A.P. Basova and co-authors [35] proposed two approaches to the study of this issue and noted that the early maturing fiber flaxes are in general taller than the late maturing linseeds. However, the earlier maturing forms among fiber flaxes, e.g., those originating from Arkhangelsk, are shorter than the genotypes from Pskov, which mature comparatively later. Similar results were obtained in Italy after an evaluation of fiber flaxes grown as winter crop [16]. The negative correlation between earliness and plant height found by N.I. Vavilov in plants at the level of various types of *L. usitatissimum* (linseed and fiber flax) is beyond doubt, but scientists still have not come to a consensus regarding the relationship between these characters within the group of fiber flaxes. Apparently, the reason is the dependency of this relationship on both the composition of the evaluated group of genotypes and the conditions of their cultivation [29]. The same applies to the relationship between earliness and seed yield, which was evaluated mainly using linseed, as well as between the yield and its components [30].

While some information about the genotypic correlations between flax characters can be found in the literature, the ecological correlations have practically not been evaluated.

The lack of data on the paratypical variability of flax traits and the relationships between them is of particular importance now in the time of tangible climate change.

The high variability of economically valuable traits of flax, the significant dependency of their manifestation on many environmental factors, as well as the lack of a reliable methodology for assessing the potential of plants at the initial stages of breeding complicate the breeding process. The main difficulties arise when determining the plant genotype by its phenotype in the course of selection for increased productivity, as well as for improvement of other quantitative traits, because they are inherited polygenically and are strongly influenced by weather conditions. Therefore, in order to increase the efficiency of breeding, it is necessary to study the indicators of continuous variability of economically valuable traits, to establish the ratio of paratypic and genotypic variation, and determine stability of their expression in various environmental conditions [36].

In addition, it becomes urgent to develop approaches to predicting stability of manifestation of economically valuable traits of varieties in changing climatic conditions.

The VIR global collection of fiber flax has been evaluated for many decades in the Pushkin Laboratories of VIR in the Northwest of Russia. At the same time, the weather conditions data were collected directly at the place of cultivation. This offers a good opportunity to reveal and clarify the genotype–phenotype relationships and the proportion of the influence of weather conditions on the studied characters, and identify the highly adaptive accessions that can be used in the further breeding process.

To achieve these goals, the variation of agronomic characters of two fiber flax varieties within 30 years of field and laboratory evaluation was compared with respective weather conditions of cultivation. The study included a search for patterns of variation in agronomic characters depending on the conditions of the year, and the ranking of 30 years of study based on the weather conditions that have a significant effect on the flax characters. It is a necessary stage for extrapolating the 30-year data to other flax varieties that have undergone a 2–3-year assessment over these years, and predicting fiber quality based on the conditions of the year.

## 2. Results and Discussion

### 2.1. Meteorological Conditions during the Study Period

**Figure 1:**
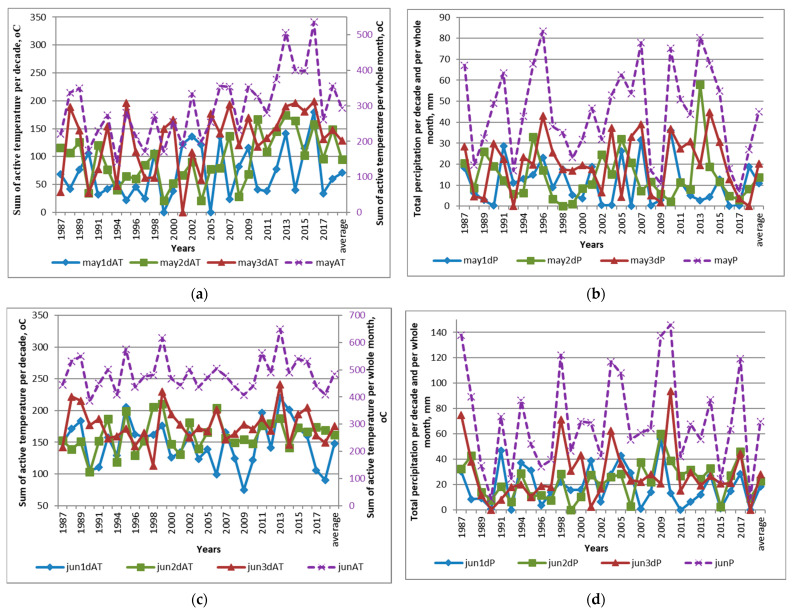

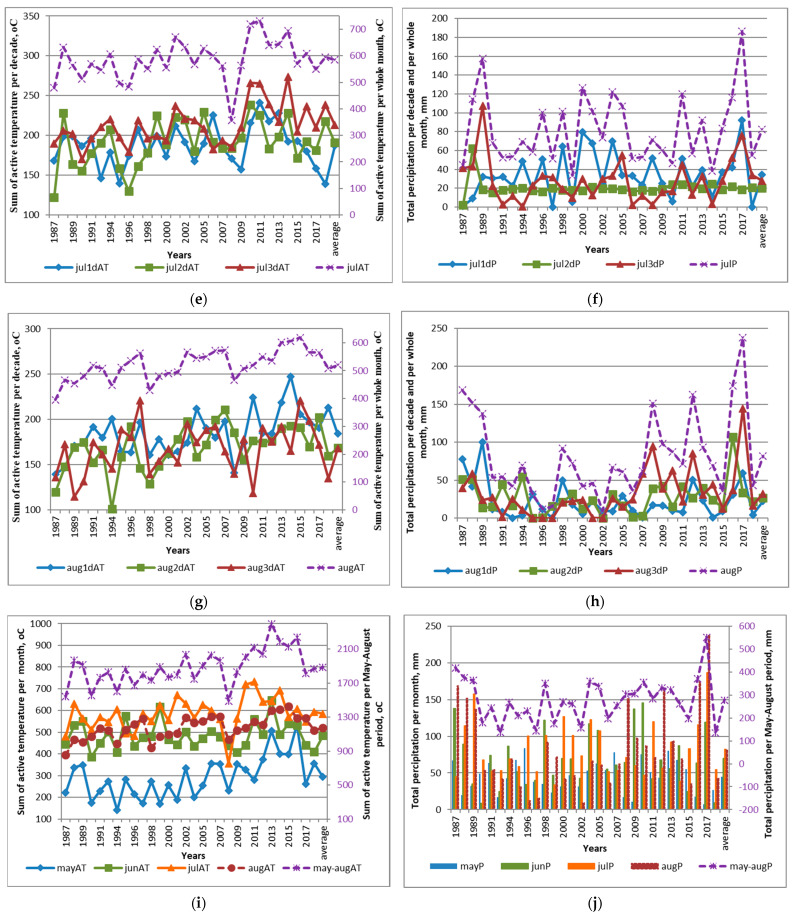

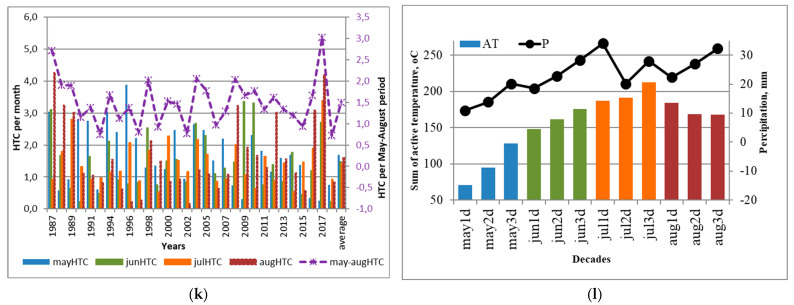
Elements of weather conditions in Pushkin in 1987–2018: (**a**) Sums of active temperatures in May and its decades. (**b**) Total precipitation in May. (**c**) Sums of active temperatures in June and its decades. (**d**) Total precipitation in June and its decades. (**e**) Sums of active temperatures in July and its decades. (**f**) Total precipitation in July and its decades. (**g**) Sums of active temperatures in August and its decades. (**h**) Total precipitation in August and its decades. (**i**) Comparisons of active temperatures per month and in the May–August period. (**j**) Comparisons of precipitation per month and in the May–August period. (**k**) Comparisons of HTC per month and in the May–August period. (**l**) Average active temperature and precipitation per decade in May–August during 30 years of observations, colors of columns indicate characters of different months of investigated.

The average temperature of May (**May**) during 30 years of evaluation was 11.9 °C (ranging from 4.5 °C in 1994 to 17.3 °C in 2016), 16.5 °C in June (**Jun**) (from 12.8 °C in 1990 to 21.6 °C in 2013), 18.8 °C in July (**Jul**) (from 11.5 °C in 2008 to 23.6 °C in 2011), and 17.0 °C in August (**Aug**) (from 12.7 °C in 1987 to 19.9 °C in 2015), and the amount of precipitation (**P**), respectively, was 44.9 mm (from 6.9 mm in 1996 to 83.4 mm in 2017), 69.7 mm (from 8.8 mm in 1990 to 145.9 mm in 2010), 82.2 mm (from 33.4 mm in 1999 to 186.5 mm in 2017) and 81.5 mm (from 9.1 in 2002 to 237.4 in 2017) (Figure 1 and Table S1).

The sum of active (>10 °C) temperatures in May was 293 °C on average (from 140 in 1994 to 536 in 2016), 485 °C in June (from 385 in 1990 to 649 in 2013), 584 °C in July (from 356 in 2008 to 762 in 2011), and 521 °C in August (from 395 in 1987 to 618 in 2015). From May to August, the sum of active temperatures averaged 1884 °C (from 1490 in 2008 to 2398 in 2013) (Table S1 and Figure 1). The highest variation coefficient for the sum of active temperatures (33%) was observed in May. In June, July and August, it was low and amounted to 13, 13 and 10%, respectively. The coefficient of variation for the amount of precipitation from May to August was high, i.e., 49% in May, 55% in June, 45% in July and 68% in August. Thus, the greatest fluctuations of temperature were observed only in May, which cannot be said about the amount of precipitation (Figure 1 and Table S1).

In general, the average air temperature during the period from 1 May till 31 August in three 10-year periods of observations was not equal. The first two decades had statistically equal average temperatures, but the third one appeared to be ~2 °C hotter than the others. These data show the tendency of global warming. At the same time, the total amount of precipitation remained at the same level. Looking ahead, we can say that this warming still has not significantly changed the expression of flax characters (data are not shown).

The hydrothermal coefficient (**HTC**) averaged 1.69 in May (from 0.26 in 2009, 2016, and 2017 to 3.89 in 1996), 1.48 in June (from 0.22 in 1990 and 2018 to 3.37 in 2009), 1.43 in July (from 0.54 in 1999 to 4.27 in 2017) and 1.61 in August (from 0.16 in 1996 and 2002 to 4.27 in 1987 and 2017), which indicated mostly sufficient and even excessive moisture. However, a decade-by-decade consideration shows that in 21 of 30 years of evaluation, conditions during the sowing time (the 1st and/or 2nd decades of May) could be characterized as insufficiently humid or even a drought (Figure 1 and Table S1). Most often, the lack of moisture has been felt in recent years.

### 2.2. Correlations between the Characteristics of Weather Conditions from 1987 to 2018 (30 Years of Evaluation)

**Figure 2:**
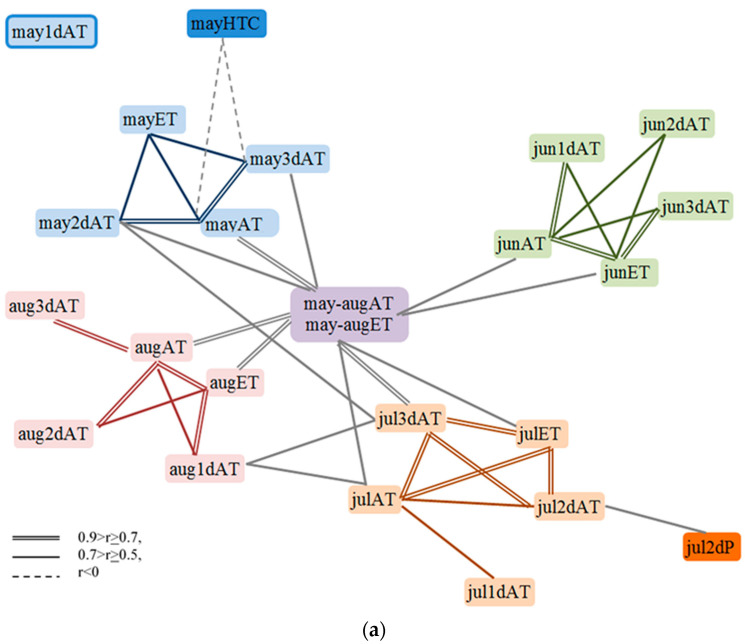

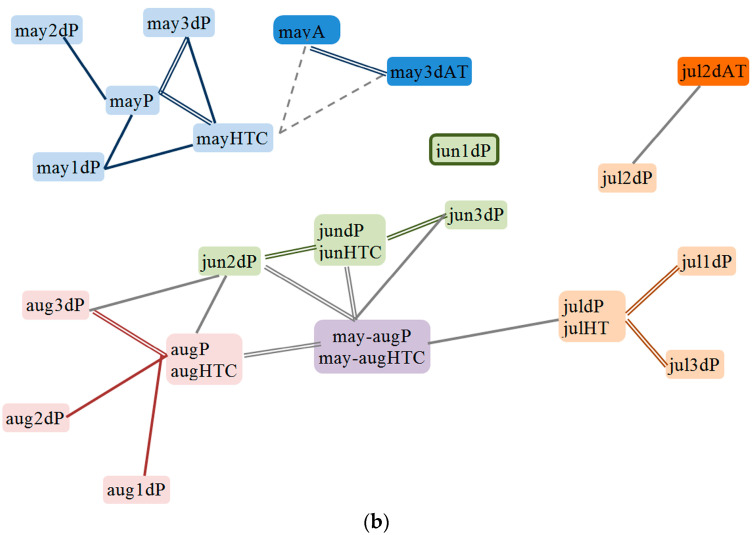
Correlations between the characteristics of weather conditions. (**a**) Correlations between air temperature characteristics in different periods. (**b**) Correlations between precipitation and HTC characteristics in different periods.

As already noted, weather conditions varied greatly depending on the year of evaluation. Correlations have been recorded between the characteristics of weather conditions, most of which relate to mathematical rather than phenological patterns (Figure 2). For instance, the sums of active temperatures for a month are closely correlated with respective sums of effective temperatures, and both of them with the cumulative temperature for the period from May to August. The total monthly active and effective temperatures are related to the active temperatures for each of the three decades. The exception is the first decade of May, which does not affect other weather characteristics. As a rule, active temperatures per decade are not related to each other even within a month; however, the hottest of them (the 2nd and 3rd decades of July and the 1st decade of August) are consistently interrelated. It is interesting that there is a connection between the temperature of the 2nd decade of May and the 3rd decade of July, but this can be explained by a strong disturbance of the normal distribution of characteristics, particularly the manifestation of maximum temperatures of these decades in 2010 and 2014. Thus, almost all temperature characteristics of different years form one pleiad with the “sum of active/effective temperatures from May to August” in the center (Figure 2). At the same time, none of the sums of effective temperatures for individual decades are correlated with other temperature characters. But this is not surprising, since the average daily temperature rarely exceeds 20 °C in this region.

The rain distribution was not so regular. Precipitation characteristics formed two pleiades: (1) of precipitation and HTC in May and (2) of precipitation and HTC in June–August, together with the total rainfall from May to August. Precipitation of all three decades in May and August correlated with the corresponding totals for the month, and the 2nd and 3rd decades of June and the 1st and 3rd ones in July with the corresponding totals for the month. Precipitation from June to August correlated with precipitation of the entire period from May to August; however, May precipitation was not associated with it. Precipitation in the 1st decade of June was independent from all other characters. The presence of a relationship between the amount of precipitation in the 2nd decade of June and the 3rd one in August is explained by the rainstorms at this time in 2009 and 2017, i.e., again, a disorder in the normal data distribution.

The hydrothermal coefficient for each month expectedly strongly correlated with the corresponding amount of precipitation. This may explain the almost identical correlations of monthly precipitation and HTC with precipitation by decade. But in May precipitation in the second decade was not directly associated with HTC.

The pleiades of precipitation and temperature are connected by negative correlations between the hydrothermal coefficient in May and the total active temperatures in May and its 3rd decade, which can be explained by abnormally high total temperatures and, accordingly, low HTC in May of 2013 and 2016. Again, a moderate correlation between the sum of active temperatures and the amount of precipitation in the 2nd decade of July is also associated with a disorder in the normal data distribution, all due to the fact that in 1987 this period was the coldest (122 °C) and the driest (1.9 mm) for the entire period of the study, and, on the contrary, in 1988 it was abnormally hot (228 °C) and wet (62.0 mm). Interestingly, the 2nd decade of July, except for these two years, was the most uniform (CV = 12%) in terms of precipitation, with 19.3 ± 0.4 mm on average.

Thus, the connections between the characteristics of weather conditions and their fluctuations can indirectly affect both economically valuable traits of flax and the connections between them, and show mathematically correct but biologically senseless connections.

### 2.3. Economically Valuable Traits of Flax and Their Variation over 30 Years of Evaluation

**Figure 3:**
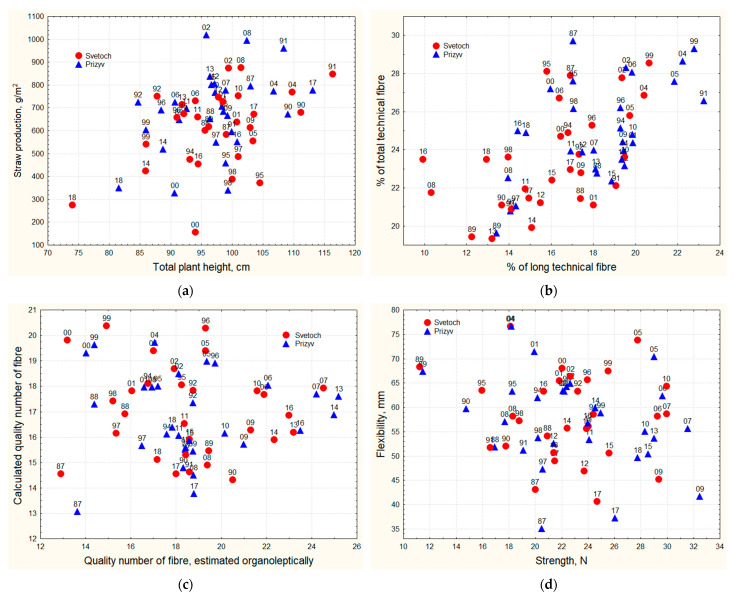

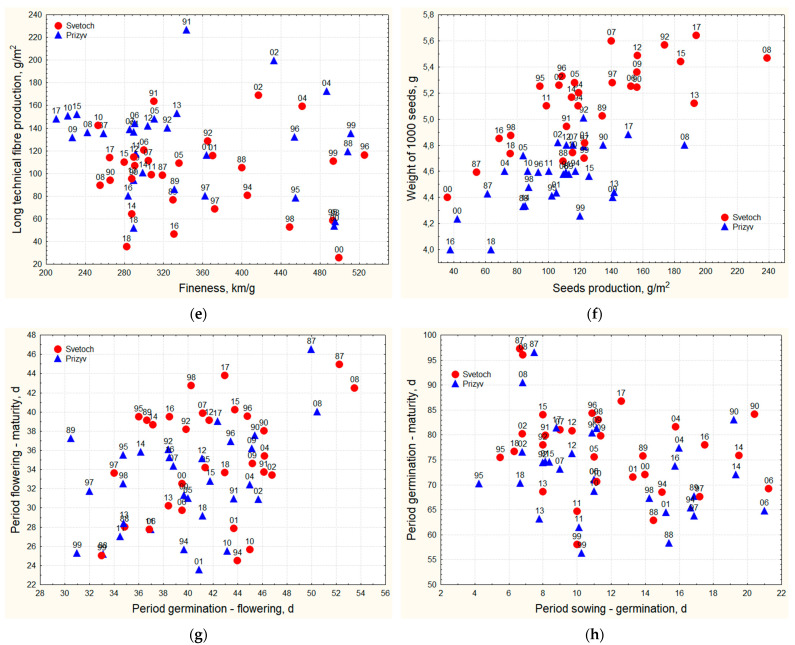
Characteristics of flax varieties Svetoch and Prizyv 81 in 1987–2018: (**a**) Total plant height and straw production. (**b**) Percentage of the total long fiber after water retting and percentage of the total technical fiber after water retting. (**c**) Quality number of long technical fiber, estimated organoleptically, and the calculated quality number of long technical fiber. (**d**) Strength of long technical fiber and flexibility of long technical fiber. (**e**) Fineness of long technical fiber and long technical fiber production after water retting. (**f**) Seed production and 1000 seed weight. (**g**) Germination-flowering and germination-maturity periods. (**h**) Sowing-germination and germination-maturity periods.

It must be mentioned that fiber flax varieties Svetoch and Prizyv 81 do not have related ancestors in their pedigrees. Both varieties do not differ significantly in plant height. During the study, the total height and technical length of the stem varied slightly (CV = 7–9%) in both varieties; these characters averaged 97 ± 1 and 83 ± 1 cm, respectively. In terms of height development, 1991 and 2017 were most favorable, when the total plant height reached 104–116 cm and technical length 90–99 cm, while 2018 was an unfavorable year, when the total height reached only 74–82 cm and technical length 63–68 cm (Figure 3 and Table S2).

In terms of straw and fiber yield, the old variety Svetoch is less productive than Prizyv 81 (**StPr** = 610 ± 32 g and 682 ± 32 g; **TF** = 143 ± 8 g and 170 ± 9 g; **LF** =99 ± 6 g and 125 ± 7 g, respectively). Straw and fiber productivity are highly variable characters (CV = 25–36%). For all three productivity indicators (straw yield, total and long fiber content) 2002 was the most favorable year: **StPr**_max_ = 875 g and 1018 g; **TF**_max_ = 241 g and 287 g; **LF**_max_ = 169 g and 226 g for Svetoch and Prizyv 81, respectively. The years 2000 and 2018 were the worst ones: **StPr**_min_ = 155 g and 327 g; **TF**_min_ = 38 g and 87 g; **LF**_min_ = 26 g and 52 g, respectively. In terms of straw yield, 1991 and 2008 were the best years; 1991 and 2004 were the best in terms of long fiber yield, while 1998 appeared to be the worst year for straw formation (Figure 3 and Table S2).

In addition to low straw productivity, the Svetoch variety has a lower fiber content, both total and long, compared to Prizyv 81 (**%TF** =23.5 ± 0.5 and 24.9 ± 0.5%; **%LF** =16.1 ± 0.5 and 18.2 ± 0.5%, respectively). These are moderately varying characters (CV = 10.1–20.3%). The year 1999 was the most favorable for an increase in formation of both total and long fiber (**%TF**_max_ = 28.6 and 29.7%; **%LF**_max_ = 20.7 and 23.2% for Svetoch and Prizyv 81, respectively), and 1989 was the worst one (**%TF**_min_ = 19.6 and 29.7%; **%LF**_min_ = 9.9 and 13.4% for Svetoch and Prizyv 81, respectively). The years 1987 and 2002 were the best for the total fiber yield, and 1991, 2004, and 2005 were the best for the long fiber formation. The worst conditions for the long fiber yield developed in 2008 and 2016 (Figure 3 and Table S2).

The two evaluated varieties do not significantly differ in fiber quality (**Str** = 23 ± 0.8 and 23 ± 0.9 N; **Flex** =58 ± 2 and 57 ± 2 mm; **Fin** =352 ± 15 and 341 ± 17 km/g; **Qo** = 18.6 ± 0.5 and 18.7 ± 0.5; **Qc** = 17.0 ± 0.3, 16.8 ± 0.3 for Svetoch and Prizyv 81, respectively). Among the fiber quality characters, the least variable ones are **Qc** and **Qo** complex indicators (CV = 10–11% and 16%, respectively). Flexibility and breaking load occupy an intermediate position with CV = 15–17% and 20–22%, respectively. Fiber fineness is a highly variable indicator (CV = 23–28%). It is impossible to determine weather conditions that would be favorable for all quality characteristics at the same time. For simple quality indicators such as the breaking load, 2006, 2007, 2009 and 2010 were most favorable years (**Str**_max_ = 30.0 and 32.5 N for Svetoch and Prizyv 81, respectively); and 1989 was the most unfavorable one (**Str**_min_ = 11.2 and 11.5 N, respectively). Fiber flexibility was the best in 2004 and 2005 (**Flex**_max_ = 77 mm for both varieties), and the worst was observed in 1987 (**Flex**_min_ = 44 and 35 mm for Svetoch and Prizyv 81, respectively). Fiber fineness was the best in 1996, 1999 and 2000 (**Fin**_max_ = 526 and 512 km/g), and the worst in 1987 (**Fin**_min_= 253 and 211 km/g) for Svetoch and Prizyv 81, respectively. The years 2007, 2013 and 2014 were the best for the complex quality indicators determined organoleptically (**Qo**_max_ = 24.5 and 25.2), while 1987, 1988, 1999, and 2000 were the worst (**Qo**_min_ = 12.9 and 13.7) for Svetoch and Prizyv 81, respectively. The most favorable weather for the calculated quality parameter was in 1996, 1999, 2000, and 2004 (**Qc**_max_ = 20.4 and 19.3), while unfavorable conditions developed in 1987 (**Qo**_min_ = 14.5 and 13.1) for Svetoch and Prizyv 81, respectively (Figure 3 and Table S2).

The average seed productivity of the Svetoch variety (126 ± 8 g) exceeds that of Prizyv 81 (105 ± 6 g). The most favorable year for this character in both varieties was 2008 (**SePr**_max_ = 239 and 187 g, respectively), and 2000 was an unfavorable one (**SeP**r_min_ = 36 and 38 g, respectively) (Figure 3 and Table S2).

Both varieties germinated simultaneously on day 12 ± 1 after sowing. However, Svetoch started flowering and matured later than Prizyv 81 (**g-f** = 42 ± 2 and 40 ± 1; f-m = 35 ± 1 and 33 ± 1; g-m = 77 ± 2 and 72 ± 2, respectively). The fastest germination (4–6 days after sowing) happened in 1995. This period was the longest in 1990, 2006, and 2014 and lasted for 19–21 days. The earliest flowering took place in 1988, 1989, 1997, and 1999 on day 33 and 31 after germination (Svetoch and Prizyv 81, respectively) and was greatly delayed in 1987 till day 54 and 50 for Svetoch and Prizyv 81, respectively. The shortest period from flowering till ripening was observed in 1988, 1989, 2001, and 2010 (24 and 25 days for Svetoch and Prizyv 81, respectively), and this period was the longest in 1987, 2008, and 2017 (47 and 44 days for Svetoch and Prizyv 81, respectively). The total flax growing season in 1988 and 1989 was shorter (58 and 56 days for Svetoch and Prizyv 81, respectively) than in other years, and the longest in 1987 and 2008 (97 days for both varieties) (Figure 3 and Table S2).

Thus, within all 30 years of evaluation, it is impossible to single out a season that is definitely the most favorable for flax development. This may be due to the complexity of positive and negative correlations formed by the economically valuable traits in variable cultivation conditions.

Also, the testing of two fiber flax varieties for 30 years has shown that the economically valuable traits react differently to the changes in weather conditions. Calculation of the variation coefficient of economically valuable traits between replications within each year and of their average values between the years showed that all of the evaluated traits can be divided into three groups.

The first group includes the most stable characters with the lowest coefficient of variation: 2.4–10.2% for the total plant height (8.5% on average over the years of study) and 2.2–11.5% for the height to inflorescence (average of 8.9%); 6.1–14.9%% for the total fiber content (11.4% on average); 1.6–6.5% for the 1000 seed weight (average of 6.3%); and 11.4% for the growing season. A small coefficient of variation of these characters, apparently, explains the significant success in flax breeding for these characters.

The second group includes the characters with medium variability, namely the long fiber content (5.9–29.0%, average of 17.2%); fiber breaking load (5.7–26.3%, average of 19.5%); fiber flexibility (4.7–16.8%, average of 15.3%); and the calculated fiber metric number (7.7–29.3, average of 23.3%).

The third group includes the highly variable characters: the yield of straw (average 28.3%), as well as the yield of long (average 36.0%) and total fiber (average 30.2%), as well as the seed yield (average 34.9%).

### 2.4. Ecological Correlations between Economically Valuable Traits

On the basis of a 30-year evaluation of the Svetoch and Prizyv 81 varieties, correlation matrices of relationships between economically valuable traits were constructed for each of them. After the z-transformation of matrices, their high similarity was proved (r = 0.96). Actually, all very strong and strong correlations (r > 0.7) in both matrices were almost identical; some of the moderate correlations (0.7 > r > 0.5) in one variety became weak (0.5 > r > 0.36) and only a few moderate ones (r < 0.52) were not found in the second variety (Table S3). It is important that during the current study, which lasted for 30 years, weather conditions were very variable, and the results revealed the potential adaptability of the tested varieties to the environmental changes. Another reason for such stability of correlation matrices may be the long breeding process and strict selection of genotypes, which give stable yield in variable weather conditions. Since the tested varieties donot have any genealogical relations, it can be supposed that the detected correlations may refer to the total group of fiber flaxes adapted to cultivation in the Non-Chernozem zone of the Russian Federation. That is why we will discuss only the correlation matrix of the Svetoch variety as the oldest one.

**Figure 4:**
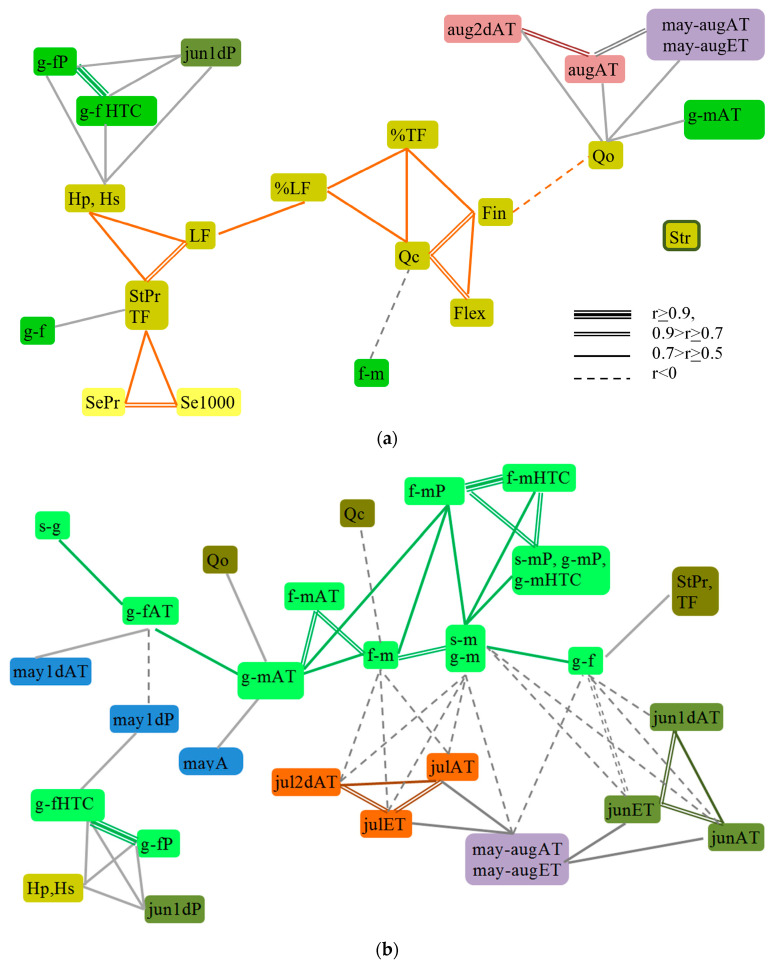
Correlations between economically valuable traits and characteristics of weather conditions. (**a**) Description of the 1st pleiade members; (**b**) Description of the 2nd pleiade members.

The economically valuable traits of flax formed three pleiades: (1) of productivity, (2) of fiber quality and yield, and (3) of the duration of growing season phases, the sums of active temperatures and precipitation during each period of plant development (Figure 4).

In the productivity pleiade, the total plant height and the height to inflorescence were closely related to each other, which is similar to the results obtained for genetic correlations in fiber flax [29,33] and linseed [30]. The straw yield expectedly very strongly correlated with the total fiber yield. On the one hand, both characters were strongly correlated with the yield of long fiber, and they were all moderately dependent on the total plant height and length of the stem to inflorescence (which coincides with genetic correlations in linseed [30]); on the other hand, they were linked with the seed productivity and 1000 seed weight. The calculated fiber quality parameter forms the center of the pleiade that includes fiber quality and yield characters. This calculated fiber quality indicator strongly depends on fiber fineness and flexibility and also is moderately linked with the long and total fiber yield, the latter two characters moderately correlating with each other. Fineness is also moderately positively correlated with flexibility and negatively with the fiber quality number (estimated organoleptically). Fiber strength is independent from other characteristics (Figure 4).

The center of the 3rd pleiade is formed by the growing season duration (from sowing and from germination to ripening), which is moderately correlated with the period from germination till flowering and strongly with the period from flowering till ripening. It is interesting to consider the relationships between duration of the growing season phases and weather conditions during these periods. It was discovered that duration of the growing season phases is moderately associated with precipitation and HTC of the corresponding periods, as well as with precipitation and HTC of the flowering-ripening period. Altogether, precipitation and HTC are strongly interconnected, which suggests that it is precipitation after flowering, rather than air temperature, that is particularly responsible for the prolongation of plant vegetation. Precipitation accumulated during the flowering-ripening period is moderately correlated with the corresponding period duration, as well as with the sum of active temperatures for the entire growing season. The sum of active temperatures during the flowering-ripening period strongly correlates with the corresponding period duration and with the sum of active temperatures for the entire growing season, which, in turn, is moderately related to the sum of active temperatures during the germination-flowering period; the latter has a moderate positive correlation with the sowing-germination phase duration. Precipitation during the period from germination till flowering strongly correlates with the HTC for the same period (Figure 4). Of course, some of these correlations can be explained by natural interdependency of the evaluated characters.

It is also interesting to consider how the weather conditions of each month affect the growing season phases. Temperatures of the 1st decade of May are positively associated with active temperatures during the germination-flowering period, which are negatively affected by precipitation of the 1st decade of May, which, in its turn, is positively associated with the HTC during the germination-flowering stage of development. The sum of active temperatures in May is moderately positively related to the sum of active temperatures during the entire growing season. However, there are no reliable correlations between the total precipitation and the sum of temperatures in May with duration of the growing season phases. That is, the weather in May does not have a direct effect on the duration of flax phenophases and other economically important traits. The sums of active temperatures in the 1st decade of June, during the whole June, and the sum of effective temperatures in June have negative correlations with the duration of the germination-flowering phase of development. The influence of effective temperatures is especially strong. For instance, the high temperatures in June, mainly during its 1st decade, accelerate the beginning of flowering. The sums of active temperatures in the 2nd decade of July, the whole July, and the sum of effective temperatures in July are negatively associated with the flowering-ripening period duration. That is, the high temperatures of July, especially its 2nd decade, play a key role in the rapid maturation. Active and effective temperatures in June and July (especially the 2nd decade of the latter), as well as active and effective temperatures from May to August, are negatively associated with the duration of ripening. Active and effective temperatures from May to August negatively correlate with the germination-flowering phase duration (Figure 4). In general, high temperatures promote plant development, while rains prolong it.

The influence of weather conditions and duration of the vegetation phases on the characters of productivity and quality of flax fiber is uncertain. Positive correlations of height to inflorescence (moderate) and total height (weak) were found to exist with precipitation and HTC during the germination-flowering phase, as well as with precipitation in the 1st decade of June, when flax is at the stage of rapid growth. The germination-flowering phase duration is moderately correlated with straw productivity and the total fiber yield. It follows from the above that the basis for flax varieties’ productivity is formed before flowering (Figure 4).

A negative relationship between the flowering-maturation phase duration and the calculated fiber quality parameter was revealed, that is, fiber quality deteriorates with the prolonged maturation period. Another complex fiber quality indicator, estimated organoleptically, is moderately correlated with active temperatures of the 2nd decade of August and of entire August; it is also influenced by the sum of active and effective temperatures from May to August and of the entire growing season. It means that plants should ripen faster after flowering for the improvement of fiber quality. Also, it is preferable that maturation occurs in the hot 2nd decade of August or earlier, if hot weather starts at the end of July (Figure 4).

### 2.5. The Influence of Conditions of the Year and of Genotype on the Expression of Morphological, Phenological and Economically Valuable Traits of Flax

**Figure 5:**
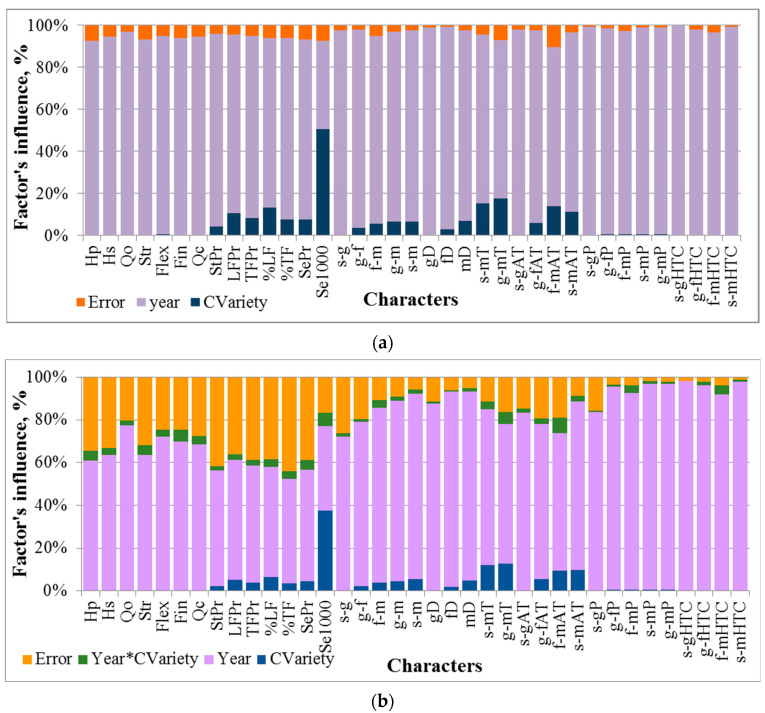
The influence of conditions of the year (Year) and genotype (CVariety) on the manifestation of morphological, phenological and economically valuable traits of flax based on the results of two-factor analysis of variance: (**a**) sampling of average values for each variety over 30 years; (**b**) sampling of several replications of each variety over 30 years. The influence of conditions of the year is significant for all characters. The influence of the variety is significant when over 0.5%.

The analysis of variance, which was carried out both for a sample of 30-year average values for each variety, and for several replications of each variety grown in one year, revealed a significant influence of conditions of a year on the manifestation of all of the evaluated characters (Figure 5). For sampling the average values of characters of a variety, the effect size of the year was almost the only one (the share of influence, ŋ^2^ > 92%) that influenced plant height, all characters of fiber quality, the sowing-germination phase duration, sums of temperatures and precipitation for this period, as well as hydrothermal coefficients for all phases of development. Since the evaluated varieties differ in the ripening time, the analysis of variance showed a significant effect of the genotype on phenological phases (4–7%), sums of active temperatures related to them (6–18%), and precipitation (~1%). The genotype also had a slight but significant effect (4–13%) on the characteristics of both fiber and seed yield, which are highly variable and depend on environmental conditions. It should be separately noted that a weakly variable and highly conservative trait—the 1000 seed weight, which, despite significant differences in environmental conditions, showed a greater dependency on the genotype (51%) than on the conditions of the year of evaluation (42%).

The ANOVA of several replications of each variety over 30 years allowed specifying the influence of the genotype and the year of evaluation on agronomic characters (Figure 5). It turned out that the interaction of conditions of the year and the genotype has a small but significant effect, especially on the characters of fiber quality (breaking load, flexibility, fineness, calculated fiber quality parameter: ŋ^2^ = 3–6%), 1000 seed weight (6%), phenophases (flowering-ripening, germination-ripening and sowing-ripening: 2–3%), the sums of temperatures (3–7%), precipitation (1–3%) and HTC (1–4%) for the germination-flowering, flowering-ripening, and germination-ripening periods. At the same time, it caused a decrease in the influence of the year conditions, whereas the random variation within the year (between replications) was quite high, from 5 to 41%.

Thus, it is necessary to find a way to rank the years in order to understand exactly what conditions are favorable for early maturation, high yield and the best quality of flax fiber.

### 2.6. Ranking the Years of Study by Weather Conditions

#### 2.6.1. Ranking the Years of Study Using the Factor Analysis

One of the most effective ways to organize the obtained results and reduce the number of analyzed characters without the loss of informativeness is the factor analysis. It allows one to identify factors that have a similar effect on groups of related characteristics of the years, and on this basis identify the years with similar weather conditions.

**Figure 6:**
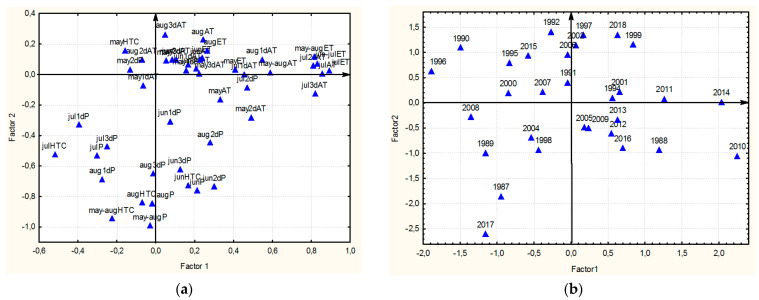

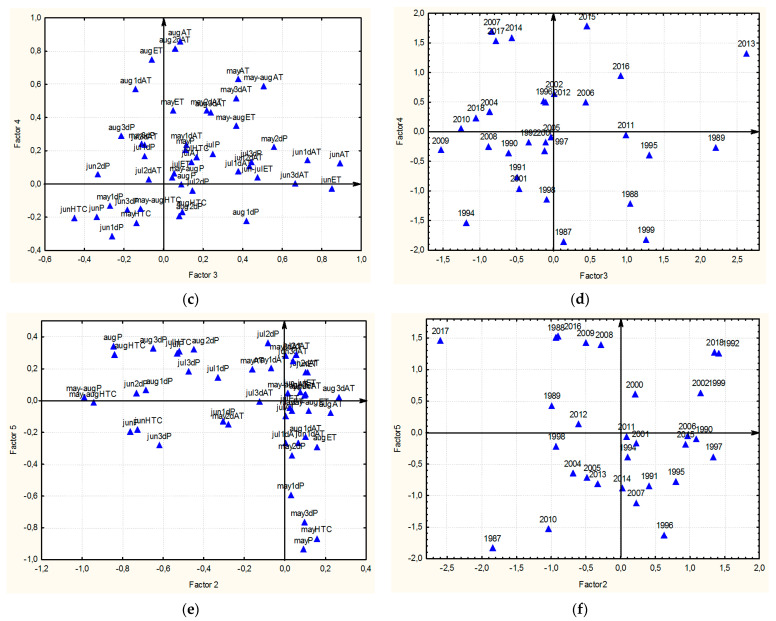
Factor loading for 45 characters and factor scores for 30 years of evaluation. (**a**) Factor 1 and Factor 2 system for flax and weather characters. (**b**) Factor 1 and Factor 2 system for the years of evaluation. (**c**) Factor 3 and Factor 4 system for flax characters. (**d**) Factor 3 and Factor 4 system for the years of evaluation. (**e**) Factor 2 and Factor 5 system for flax characters. (**f**) Factor 2 and Factor 5 system for the years.

Seven factors responsible for 66% of the total variation among the characters were identified using the scree test (Figure S1). Initial results of the factor analysis could not be uniquely interpreted, as many characters had great loads of several factors (data are not shown). Consequently, the varimax rotation of the axes (raw varimax) was carried out to reach the simplest elementary structure (Figure 6 and Tables S4 and S5).

**The first factor** (accounts for 16% of variation) can be interpreted as heat in July–early August, and during the growing season. It determines the high loads on the sum of active temperatures for each decade of July and the total month, as well as the 1st decade of August, the sum of effective temperatures in July, June–July, and during the growing season. Also, this factor is positively associated with the amount of precipitation in the 2nd decade of July and negatively with the 1st decade of the same month and the HTC of July (Figure 6 and Table S4).

The lowest loads of the 1st factor were recorded in 1987, 1989, 1990, 1996, and 2008, i.e., it was cold during the flowering and maturation of flax in July and early August, and the highest loads were recorded in 1988, 2010, 2011, and 2014, which were hot during these periods (Figure 6 and Table S5).

**The second factor** (accounts for 17% of variation) can be interpreted as a drought from June to August, as it characterizes the minimum amount of precipitation in each decade of and in entire June and August, as well as in the 3rd decade of and entire July, and also low HTC from June to August and totally for the entire growing season (Figure 6 and Table S4).

For this factor, the lowest loads were recorded in 1987, 1988, 1989, and 2017, which were rainy from June to August, during the period from rapid growth to ripening of flax, and the greatest loads were recorded in the driest years, i.e., 1990, 1992, 1997, 1999, 2002, 2006, and 2018 (Figure 6 and Table S5).

**The third factor** (accounts for 12% of variation) can be interpreted as the heat in June, which determines the high active temperatures in each decade of and in entire June, as well as the sum of active temperatures from May to August and effective temperatures in June. The amount of precipitation in the 2nd decade of May is also associated with this factor (Figure 6 and Table S4).

For this factor, the lowest loads were recorded in 1994, 2009, and 2010 with a cold June, which, as a rule, encompasses the herringbone stage, rapid growth and budding, and the greatest loads were recorded in 1989 and 2013, i.e., the years with the hottest June (Figure 6 and Table S5).

**The fourth factor** (accounts for 11% of variation) can be interpreted as the heat in mid-late May and in August. It characterizes the high active temperatures in the 2nd and 3rd decades and their total in May, in each decade and the totals of August, the totals from May to August, as well as the effective temperatures of May and August (Figure 6 and Table S4).

For this factor, the lowest loads were recorded in 1987, 1994, and 1999, with cold May (seedlings and herringbone stages) and August (ripening), and the greatest loads were recorded in 2007, 2013, 2014, 2015, and 2017, which are characterized by hot May and August (Figure 6 and Table S5).

**The fifth factor** (accounts for 10% of variation) determines the drought in May—low precipitation in all decades and in entire May, as well as low HTC during this period (Figure 6 and Table S4).

According to this factor, the lowest loads were recorded in 1987, 1996, and 2010, with rainy May, falling on the seedlings-herringbone stage period, and the greatest loads were recorded in 1988, 1992, 2008, 2009, 2016, 2017, and 2018, which were the driest years (Figure 6 and Table S5).

The factor analysis resulted in determining 10 groups of years. Also, many years of evaluation were included in two or more groups, and 1991, 1995, 1998, 2000, 2001, 2004, 2005, and 2012 showed reliable, but not maximum, factor loads.

Thus, it is not possible to use the factor analysis directly to classify the years. Therefore, based on the results of the factor analysis, the K-means cluster analysis (Euclidian distance) was used, which showed that the minimum number of clusters combining all five factors and 30 years of evaluation is 7.

#### 2.6.2. Ranking the Years of the Study Using the Cluster Analysis Based on the Results of the Factor Analysis

Since the factor analysis could not directly and clearly separate the years of evaluation into groups, we carried out the next stage of classifying the years. For this purpose, we used the factor loads, which had been obtained on the basis of the factor analysis results. They were used as characters for the K-means cluster analysis, which makes possible an unambiguous division into groups, indicating the reliability of the inclusion of each character (in our case, a factor) in the group. Also, this cluster analysis at the next analytical stage determines the contingent center of each cluster and, relative to it, creates the distance to each year in the cluster. Thus, the analysis shows the number of clusters and their relative positions, as well as the position of the year of evaluation within each cluster.

The analysis of variance showed that all five factors affect the resulting tree. The 3rd factor has the most influence, and the 4th factor has the least influence (Table S6).

**Figure 7:**
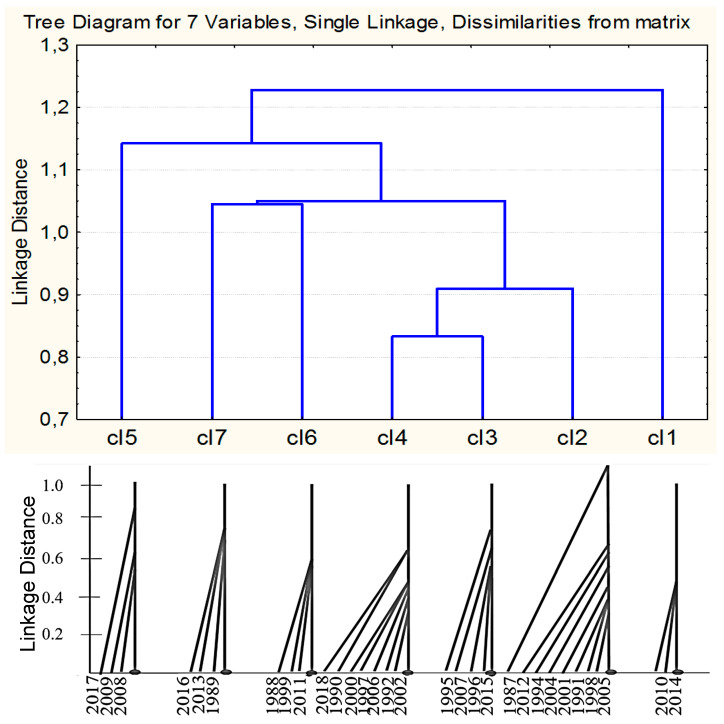
Clustering of the years of flax evaluation based on the results of the factor and cluster analyses (K-means).

**Figure 8:**
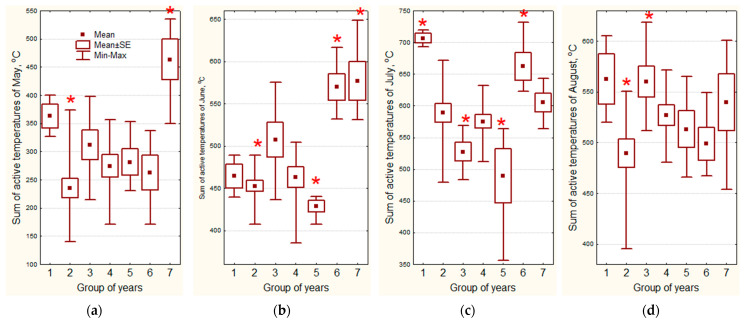

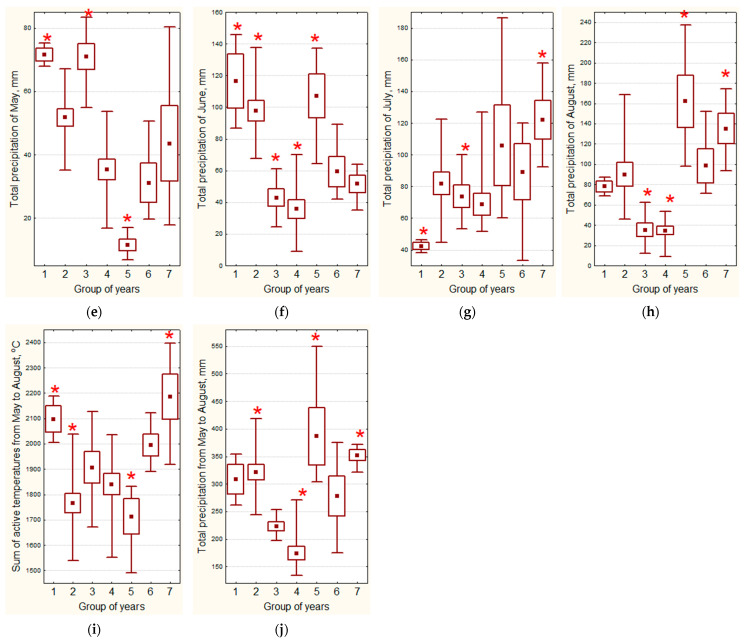
Characteristics of the years grouped by the sums of active temperatures and precipitation. Asterisk marks the groups which significantly differ from the others: (**a**) the sum of active temperatures of May; (**b**) the sum of active temperatures of June; (**c**) the sum of active temperatures of July; (**d**) the sum of active temperatures of August; (**e**) total precipitation in May; (**f**) total precipitation in June; (**g**) total precipitation in July; (**h**) total precipitation in August; (**i**) the sum of active temperatures from May to August; (**j**) total precipitation from May to August.

**The first cluster** is formed by the years with heat in May, July, August, cold in June and rains in May. It includes 2010 and 2014, which are characterized by the maximum factor loads for factors 1 and 4, as well as the minimum for factors 3 and 5 (Figure 7 and Figure 8 and Table 1).

**The second cluster** is the largest one; it is formed by the years with cold May and August and rains in May, June, July, August and in the growing season as a whole. It includes 1987, 1991, 1994, 1998, 2001, 2004, 2005 and 2012, which are characterized by the minimal factor loads for factors 2, 4 and 5 (Figure 7 and Figure 8 and Table 1).

**The third cluster** is formed by the years with heat in May and August, cold in July, rains in May, and drought in June, July, August, and in the growing season as a whole. It includes 1995, 1996, 2007 and 2015, which are characterized by the maximum factor loads for factors 2 and 4, as well as the minimum for factors 1 and 5 (Figure 7 and Figure 8 and Table 1).

**The fourth cluster**, the second largest one, is formed by the years with drought in May, June, July and August, i.e., during the entire growing season. This cluster includes 1990, 1992, 1997, 2000, 2002, 2006 and 2018, which are characterized by the maximum factor loads for factors 2 and 5 (Figure 7 and Figure 8 and Table 1).

**The fifth cluster** is formed by the years with cold June, July and the entire growing season, with a drought in May, and rains in June, July, August, and in the growing season as a whole. This cluster includes 2008, 2009 and 2017, which are characterized by the maximum factor loads for factor 5, as well as the minimum for factors 1, 2 and 3 (Figure 7 and Figure 8 and Table 1).

**The sixth cluster** is formed by the years with cold May and August, heat in June, July and the entire growing season, and drought in May. This cluster includes 1988, 1999 and 2011, which are characterized by the maximum factor loads for factors 1 and 3, as well as the minimum for factor 4 (Figure 7 and Figure 8 and Table 1).

**The seventh cluster** is formed by the years with hot May, June and August and rains in June, July, August, and during the whole growing season. This cluster includes 1989, 2013 and 2016, which are characterized by the maximum factor loads for factors 3 and 4, as well as the minimum loads for factors 2 and 5 (Figure 7 and Figure 8 and Table 1).

Clusters 3 and 4 are the closest to each other, while cluster 1 is the most isolated one (Figure 7).

Thus, according to the results of the joint factorial and cluster analyses, it was possible to unambiguously classify all of the years of evaluation according to weather conditions.

### 2.7. Dependency of Economically Valuable Traits on the Grouping of Years into Clusters

**Figure 9:**
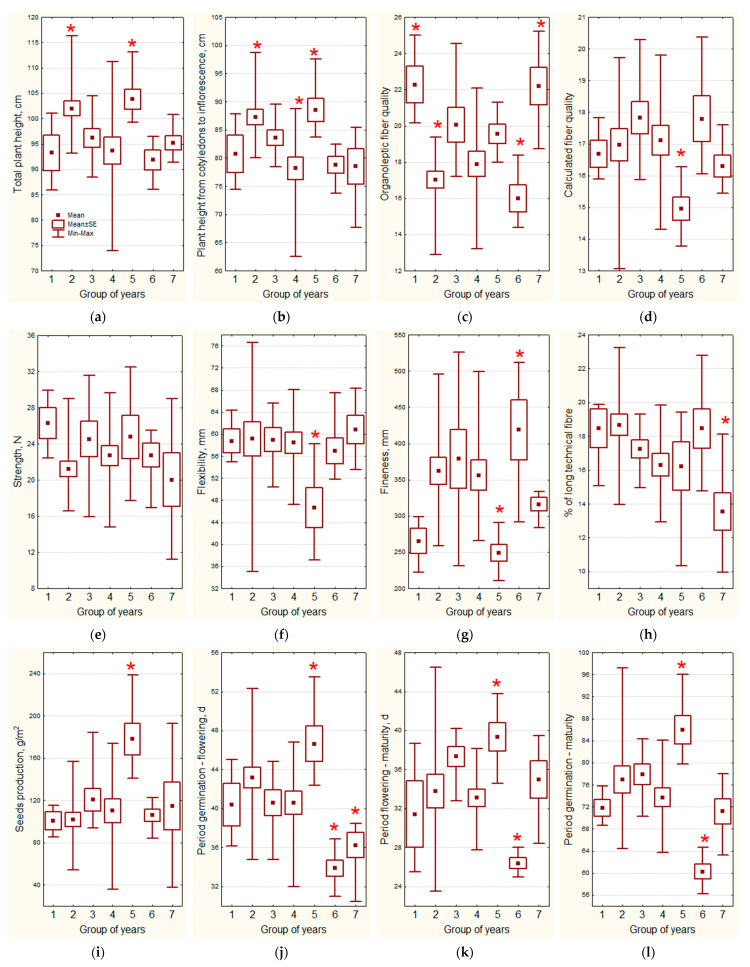
Characteristics of groups of years by economically valuable traits. Asterisk marks the groups which significantly differ from the others: (**a**) total plant height; (**b**) plant height from cotyledons to inflorescence; (**c**) quality number of long technical fiber, estimated organoleptically; (**d**) the calculated quality number of long technical fiber; (**e**) strength of long technical fiber; (**f**) flexibility of long technical fiber; (**g**) fineness of long technical fiber; (**h**) % of long technical fiber extracted after water retting; (**i**) seed production; (**j**) germination-flowering period; (**k**) flowering-maturity period; (**l**) germination-maturity period.

The seven obtained clusters reflect the entire spectrum of weather conditions observed over 30 years of evaluation. Weather conditions could both positively and negatively affect the manifestation of economically valuable traits. Using the Mann–Whitney U test and Student’s t-criteria, it is possible to identify significant differences between the years from the cluster being compared with the rest of the analyzed years (Table 2 and Figure 9).

Weather conditions of 2010, 2014, which form the **first cluster** (hot May, July, August, cold June, rains in May), caused very early sowing (12 days earlier than the average for all years) and led to speeding up the development in the phases of germination, flowering and maturation, which positively affected the organoleptically estimated fiber quality (22 ± 1 compared to overall average value of 18 ± 0).

Weather conditions of 1987, 1991, 1994, 1998, 2001, 2004, 2005 and 2012, which form the **second cluster** (cold May and August, rains in May, June, July, August, and in the growing season as a whole) had a positive effect on the total plant height (104 ± 2 compared to the overall average of 96 ± 1 cm), and height to inflorescence (89 ± 2 compared to the overall average of 82 ± 1 cm), but negatively affected the organoleptically estimated fiber quality (17 ± 0 compared to the overall average of 19 ± 0).

Weather conditions in 1995, 1996, 2007 and 2015, which form the **third cluster** (heat in May and August, cold in July, rains in May, drought in June, July, August, and in the entire growing season) did not have a significant impact on the economically valuable traits of flax.

Weather conditions of 1990, 1992, 1997, 2000, 2002, 2006 and 2018, which form the **fourth cluster** (lack of moisture in the entire growing season) negatively affected only the height to inflorescence (78 ± 2 compared to the overall average of 84 ± 1 cm), possibly because the drought was not severe and precipitation occurred, although in small amounts.

Weather conditions in 2008, 2009 and 2017, which form the **fifth cluster** (cold June, July and the growing season as a whole, lack of precipitation in May, rains in June, July, August, and in the growing season as a whole) slowed down the passage of all phases of flax development, which positively affected the total height (104 ± 2 compared to the overall average of 96 ± 1 cm), height to inflorescence (89 ± 2 compared to the overall average of 82 ± 1 cm) and seed productivity (178 ± 15 g compared to the overall average of 109 ± 5 g). However, these conditions had an extremely negative impact on such fiber quality parameters as flexibility (47 ± 4 compared to the overall average of 59 ± 1 mm), fineness (249 ± 12 compared to the overall average of 357 ± 12 km/gr) and the calculated fiber quality (15 ± 0 compared to overall average 17 ± 0).

Weather conditions in 1988, 1999 and 2011, which form the **sixth cluster** (cold May and August, hot June, July, and the growing season as a whole, drought in May) led to a shortening of all phases of flax development for a week or more, i.e., to 34 ± 1 days of germination-flowering period compared to the overall average of 42 ± 1 days; 26 ± 1 days of flowering-ripening period compared to the overall average of 35 ± 1 days; 60 ± 1 days of the growing season compared to the overall average of 76 ± 1 days, and to harvesting 11 days earlier than the average, which had an extremely positive effect on the fiber fineness (419 ± 41 compared to the overall average of 338 ± 11 km/gr), but negatively influenced the organoleptically estimated fiber quality (16 ± 0 compared to the overall average of 19 ± 0).

Weather conditions in 1989, 2013 and 2016, which form the **seventh cluster** (hot May, June and August, rains in June, July, August, and in the growing season as a whole) led to a shortening of the germination-flowering period (36 ± 1 compared to the overall average of 41 ± 1 days), which negatively affected the output of long fiber (14 ± 1% compared to the overall average of 18 ± 1%), which, however, was of better quality determined organoleptically (22 ± 1 compared to the overall average of 18 ± 0).

Thus, for the first time, we conducted the ranking and grouping of the years of evaluation and assessed their impact on the economically valuable traits.

So, hot weather at the beginning of the growing season, which causes early germination, leads to a significant increase in the organoleptically estimated fiber quality; drought leads to a decrease in technical length; cool and rainy weather during the period starting with rapid growth, prolongs the duration of vegetation phases and increases plant height and seed productivity, but degrades the flexibility and fineness of the fiber; the cold in May and the heat in June and July accelerate the passing of the growing season phases and improve fiber fineness, but degrade its organoleptic parameters.

Consequently, the proposed methodology will help to classify and select other flax accessions already evaluated in 1987–2018, taking into account the appropriate weather conditions of the year, without additional testing.

## 3. Materials and Methods

Field experiments were carried out in one field (with crop rotation) in the Leningrad Province of the Russian Federation. It is located at 60 N near the Baltic Sea. In June, the daylight reaches 20 h. The average temperature during the growing season of flax is 14 °C, and the total precipitation reaches 350 mm on average. Soils of this region are classified as brown podzolic light loams with humus content of 3–4% and pH 5.5–6.0. These conditions are very favorable for fiber flax cultivation.

Meteorological observations during the years of the study were carried out at a meteorological station located directly on the experimental field. The air temperature was recorded every hour, and the average daily air temperature was calculated. The amount of precipitation was recorded daily. The sums of daily active temperatures (**AT**) (>10 °C) and precipitation (**P**) (mm) were calculated separately for each decade (**1d**, **2d**, **3d**) and the whole month from May to August, as well as the sums of effective temperatures (**ET**) (>20 °C). The monthly level of humidity in the territory was also expressed by the Selyaninov hydrothermal coefficient (**HTC**), which was calculated as a ratio between the total precipitation in mm during the period with daily mean air temperatures above 10 °C, and the sum of temperatures for the same period of time reduced tenfold [37]. For fiber flax, **HTC** values above 1 are necessary. A coefficient above 1.6 means that the humidity is excessive [38].

For the current work, the results of fiber flax field evaluation from 1987 till 2018 were taken, except for 1993 and 2003, which turned out to be extremely unfavorable for flax. All of the straw rotted in the field, and that is why the technical analysis of the fiber was not carried out. Thus, the total period of flax evaluation presented in this article was 30 years.

The sums of active temperatures, precipitation and hydrothermal coefficient for each of the main phases of plant development of both varieties (sowing-germination, germination-flowering, flowering-ripening, germination-ripening), as well as the sum of temperatures for the entire growing season from sowing and from germination to ripening, were calculated separately.

For the current work, we selected two standard varieties used for the evaluation of the VIR fiber flax collection, namely Svetoch (Russia, Torzhok, the All Russian Institute of Flax, k-5333) and Prizyv-81 (Belarus, Mogilev Experimental Station, k-7472). The Svetoch variety has been used for evaluating the VIR collection from 1954 to present, which allows a comparison of the data on the VIR collection testing obtained during this period of time, and the variety Prizyv-81 has been designated as the standard for earliness since 1987. The article analyzes the results of these varieties’ field evaluations from 1987 to 2018.

The tested flax varieties were sown annually in 2–22 replications. The sowing date varied greatly depending on the weather conditions of the year. During the described period, the earliest sowing was carried out on 23 April in 1990 and 2004, and the latest one on 24 May in 1997 (Table S2).

Flax was sown on 1 m^2^ plots. The seeding rate was 2000 seeds/m^2^. The rows were spaced at 8 cm. The field evaluation of the varieties was carried out in accordance with the Methodological Guidelines [39]. It included registration of the periods from sowing to germination (of 75% of seeds, **s-g**), from germination to full flowering (of 75% of plants, **g-f**), from full flowering to early yellow ripening of half of the bolls in the plot (**f-m**), from germination to maturity (**g-m**), from sowing to maturity (**s-m**), as well as the total plant height (**Hp**), height to inflorescence (**Hs**), seed yield in g/m^2^ (**SePr**), 1000 seed weight (**Se1000**) and straw production in g/m^2^ (**StPr**).

Harvesting was carried out at the stage of early yellow ripening. Fiber was extracted by water retting at 32–37 °C and pH ≤ 2.5 for 3 days. Fiber content and quality were evaluated according to the standard method accepted in the Russian Federation [40]. To determine the fiber content in stems, the straw was first dried to a constant weight, and 100 g of dry straw was taken from each sample for testing. The stock was broken and scutched. The total fiber content (**%TF**) was calculated relative to the straw weight. Fibers were hackled to separate long fibers, and their content (**%LF**) was calculated relative to the straw weight. After this, long fiber quality was tested. For instrumental measurements of fiber flexibility (**Flex**), special fiber preparations of 30 individual strands were made. A 27 cmlong sample weighing 420 mg was cut from the middle of each fiber strand. All preparations were flattened under the press for 8–10 h at 20 °C and humidity of 60–65%. Fiber flexibility (mm) was measured according to the free flexure of both strand ends.

The fiber breaking load (Newtons) was measured with a dynamometer using the same 30 individual strands (27 cm long, 420 mg) for each sample. It must be mentioned that the data on the technical fiber breaking load allow the estimation of comparative differences between the samples in terms of the technical fiber strength (**Str**), but give no information about the strength of elementary fibers.

For the measurement of fiber fineness (**Fin**), a fiber strand was carded on a special hatchel (10 needles per 1 cm). A 10 mmlong part was cut out from the middle of the strand. Five 10 mg preparations were taken to count the number of fibers (n) in each preparation. Then, the average number of fibers per preparation was calculated. The average length of 1 mg of fiber was taken as (10 mm × n/10). Finally, the fineness was estimated in km/g.

Two general quality characters of technical fiber samples were **Qo** estimated organoleptically by experts, taking into account the length, color, brightness, fineness, flexibility, and strength, and **Qc** calculated from the three described parameters according to Formula (1).

***Qc*** = 0.2 × ***Str*** + 0.1 × ***Flex*** + 0.013 × ***Fin*** + 2.1 (1)

All characters (in bold in the text) used in the current study or for statistical analyses are listed in Table 3.

Mathematical processing of the obtained results was carried out by standard statistical methods [32,41,42,43,44]. The influence of the genotype and conditions of the year on the expression of flax characters was evaluated using the two-factor analysis of variance in the Statistica 7.0 program [44]. The effect size of the factor influence was estimated by Fisher’s Formula (2).

***ŋ*^2^** = ***SSfactor/SStotal*** × 100% (2)

The variation coefficients (CV) were calculated using the standard formula within the character expression during 30 years for each of the varieties independently. They were classified as low at CV ≤ 10%, intermediate at 25% ≥ CV > 10%, and high at CV > 25% [32].

Using the data obtained during 30 years of evaluation of the same two flax varieties, ecological correlations between their characters (Pearson correlation coefficients) were calculated according to the standard methods [32,41,42,43,44]. Calculations were made with the use of MS Excel and Statistica 7.0 for Windows analytical packages. For n = 30 and *p* = 0.05, r > 0.36 was considered as significant, 0.7 > r ≥ 0.5 as intermediate, 0.9 > r ≥ 0.7 as high, and r ≥ 0.9 as very strong correlations. The correlation pleiads were drawn manually [32]. The similarity of different correlation matrices was evaluated using the Fisher z-transformation [32].

Factor analysis was also carried out. The principal component method of analysis was used to extract the factor load. “The rocky scree” method was used to identify the number of factors (Scree plot). To obtain the simplest structure of factor loadings, the factor rotation method (Varimax raw) was used [32,42,44]. Factor scores were used for line classification. This method allows classifying the years not by individual weather indicators, but by the most interrelated weather conditions. Among all of the possibilities of pair-factor systems, four pictures were chosen in order to show all five factors and indicate the most evident grouping of characters. After this, cluster analysis (K-means method with Euclidian distance calculation) was used for the complete classification of lines [32,42,44].

## 4. Conclusions

Meteorological observations carried out in 1987–2018 in the Northwest of Russia showed that the third decade of this period was ~2 °C hotter.

Weather has a significant impact on the economically valuable flax traits, ranging from 40 (1000 seed weight) to 80% (fiber quality).

Weather conditions in May do not have a direct effect on the economically important traits. High temperatures in June accelerate flowering. Hot weather in July plays a key role in the rapid maturation. An increase in temperature promotes plant development, while rains prolong it.

Plants height is positively affected by precipitation during the germination-flowering period and in the 1st decade of June. The germination-flowering phase is correlated with straw productivity and total fiber yield. That is, the basis for the productivity of flax varieties is formed before flowering. High-quality fiber is formed in hot conditions from the end of July till mid-August. Under prolonged maturation, fiber quality deteriorates.

To obtain high yield of the best quality fiber, it is preferable to plant flax early in spring and use late-flowering and fast-ripening varieties.

The suggested method for classifying meteorological conditions of a year can be used in other genebanks for systematizing and analyzing the results of field evaluation of crops.

## Figures and Tables

**Table 1:** Characteristics of seven clusters obtained using the K-means method based on the results of the factor analysis.

Cluster	Cluster Characters	Cluster Member	Distance From Respective Cluster Center	Descriptive Statistics for Cluster (Factor Mean + Sd)				
				Factor 1	Factor 2	Factor 3	Factor 4	Factor 5
1	Hot weather in May, July, August; cold in June; rains in May	2010	0.47	2.14 ± 0.15 *	−0.51 ± 0.75	−0.91 ± 0.48	0.82 ± 1.10	−1.20 ± 0.46
		2014	0.47					
2	Cold weather in May, August; rains in May, June, July, August and the growing season	1987	1.09	0.00 ± 0.59	−0.48 ± 0.73	−0.39 ± 0.46	−0.69 ± 0.86	−0.58 ± 0.60
		1991	0.42					
		1994	0.64					
		1998	0.41					
		2001	0.49					
		2004	0.57					
		2005	0.33					
		2012	0.68					
3	Hot weather in May and August; cold in July; rains in May; drought in June, July, August and the growing season	1995	0.76	−0.92 ± 0.67	0.65 ± 0.31	0.20 ± 0.90	0.90 ± 1.03	−0.92 ± 0.61
		1996	0.58					
		2007	0.66					
		2015	0.56					
4	Drought in growing season	1990	0.65	−0.25 ± 0.71	1.08 ± 0.42	−0.25 ± 0.48	0.05 ± 0.41	0.46 ± 0.66
		1992	0.40					
		1997	0.47					
		2000	0.49					
		2002	0.33					
		2006	0.45					
		2018	0.66					
5	Cold weather in June, July and the growing season; drought in May; rains in June, July, August and the growing season	2008	0.54	−0.76 ± 0.87	−1.13 ± 1.28	−1.06 ± 0.41	0.33 ± 1.05	1.43 ± 0.03
		2009	0.63					
		2017	0.88					
6	Cold weather in May, August; hot weather in June, July and the growing season; drought in May	1988	0.60	1.10 ± 0.22	0.10 ± 1.04	1.10 ± 0.14	−1.03 ± 0.90	0.70 ± 0.79
		1999	0.60					
		2011	0.56					
7	Hot weather in May, June, August; rains in June, July, August and the growing season	1989	0.71	0.06 ± 1.05	−0.74 ± 0.35	1.92 ± 0.89	0.67 ± 0.83	0.38 ± 1.18
		2013	0.76					
		2016	0.75					

* intensity of green colors indicate the minimum value intensity of the red colors indicate the maximum value intensity.

**Table 2:** Comparison of economically valuable traits of Svetoch and Prizyv 81 varieties grown in different years according to Mann–Whitney U test and Student’s *t*-criteria.

Characters	Cluster Characters						Criterion			
	Compared Cluster			Other Clusters			Mann–Whitney U		Student’s *t*	
	N	Mean ± Se	RankSum	N	Mean ± Se	RankSum	Z Adjusted	*p*-Level	*t*-Value	*p*
**Cluster 1 (2010, 2014)**										
**Qo**	4	22 ± 1 *	208	56	18 ± 0	1622	2.55	0.01	2.70	0.0090
**sD**	4	25/4 ± 1	20	56	7/5 ± 1	1810	−3.03	0.0024	−3.81	0.0003
**gD**	4	10/5 ± 1	28	56	19/5 ± 1	1802	−2.79	0.0053	−2.69	0.0094
**fD**	4	20/6 ± 1	21	56	29/6 ± 1	1809	−2.99	0.0028	−3.32	0.0016
**mD**	4	21/7 ± 2	33	56	2/8 ± 1	1797	−2.64	0.0083	−2.70	0.0091
**Cluster 2 (1987, 1991, 1994, 1998, 2001, 2004, 2005, 2012)**										
**Hp**	16	102 ± 1	671	44	95 ± 1	1159	3.06	0.0022	3.12	0.0028
**Hs**	16	87 ± 1	658	44	81 ± 1	1172	2.84	0.0045	3.31	0.0016
**Qo**	16	17 ± 0	322	44	19 ± 0	1508	−2.78	0.0055	−2.74	0.0082
**Cluster 3 (1995, 1996, 2007, 2015)—no difference**										
**Cluster 4 (1990, 1992, 1997, 2000, 2002, 2006, 2018)**										
**Hs**	14	78 ± 2	288	46	84 ± 1	1542	−2.43	0.02	−2.87	0.0058
**Cluster 5 (2008, 2009, 2017)**										
**Hp**	6	104 ± 2	293	54	96 ± 1	1537	2.71	0.0067	2.29	0.03
**Hs**	6	89 ± 2	283	54	82 ± 1	1548	2.45	0.01	2.25	0.03
**Flex**	6	47 ± 4	76	54	59 ± 1	1754	−2.64	0.0084	−3.36	0.0014
**Fin**	6	249 ± 12	47	54	357 ± 12	1783	−3.35	0.0008	−3.07	0.0032
**Qc**	6	15 ± 0	64	54	17 ± 0	1766	−2.93	0.0034	−3.12	0.0028
**SePr**	6	178 ± 15	326	54	109 ± 5	1504	3.52	0.0004	4.75	0.0000
**g-f**	6	47 ± 2	293	54	40 ± 1	1538	2.70	0.0070	3.16	0.0025
**f-m**	6	39 ± 1	288	54	33 ± 1	1542	2.59	0.0097	2.62	0.01
**g-m**	6	86 ± 3	310	54	73 ± 1	1520	3.13	0.0018	3.52	0.0008
**s-m**	6	96 ± 2	295	54	85 ± 1	1535	2.76	0.0058	2.80	0.0070
**Cluster 6 (1988, 1999, 2011)**										
**Qo**	6	16 ± 1	80	54	19 ± 0	1750	−2.54	0.01	−2.44	0.02
**Fin**	6	419 ± 41	266	54	338 ± 11	1564	2.05	0.04	2.21	0.03
**g-f**	6	34 ± 1	44	54	42 ± 1	1786	−3.43	0.0006	−3.83	0.0003
**f-m**	6	26 ± 1	45	54	35 ± 1	1786	−3.41	0.0006	−3.79	0.0004
**g-m**	6	60 ± 1	24	54	76 ± 1	1806	−3.92	0.0001	−4.78	0.0000
**s-m**	6	72 ± 2	29	54	88 ± 1	1801	−3.80	0.0001	−4.94	0.0000
**mD**	6	22/7 ± 1	55	54	2/8 ± 1	1775	−3.15	0.0016	−3.08	0.0032
**Cluster 7 (1989, 2013, 2016)**										
**Qo**	6	22 ± 1	306	54	18 ± 0	1525	3.02	0.0025	3.40	0.0012
**%LF**	6	14 ± 1	66	54	18 ± 0	1764	−2.88	0.0039	−3.50	0.0009
**g-f**	6	36 ± 1	85	54	41 ± 1	1746	−2.43	0.02	−2.36	0.02

* green colors indicate the minimum value, of the red colors indicate the maximum value.

**Table 3:** Designations and descriptions of flax and year characters.

Designation	Characters
cv	Commercial variety
	**Date**
year	Sowing year, year
sD	Sowing date, day/month
gD	Germination date, day/month
fD	Flowering date, day/month
mD	Maturity date, day/month
	**Growing season period**
s-g	Sowing-germination period, d
g-f	Germination-flowering period, d
f-m	Flowering-maturity period, d
g-m	Germination-maturity period, d
s-m	Sowing-maturity period, d
	**Productivity**
Hp	Total plant height, cm
Hs	Plant height from cotyledons to inflorescence, cm
StPr	Straw production, g/m^2^
LFPr	Long technical fiber production after water retting, g/m^2^
TFPr	Total technical fiber production after water retting, g/m^2^
%LF	% of long technical fiber after water retting, %
%TF	% of total technical fiber after water retting, %
SePr	Seed production, g/m^2^
Se1000	1000 seed weight, g
	**Fiber quality**
Str	Strength of long technical fiber, N
Flex	Flexibility of long technical fiber, mm
Fin	Fineness of long technical fiber, km/g
Qo	Quality number of long technical fiber estimated organoleptically
Qc	Calculated quality number of long technical fiber (0.2 × **Str** + 0.1 × **Flex** + 0.0013 × **Fin** +2.1)
	**Sum of temperatures for the growing season**
s-mT	Sum of temperatures from sowing to maturity, °C
g-mT	Sum of temperatures from germination to maturity, °C
	**Sum of active temperatures (>10 °C) for the growing season**
s-gAT	Sum of active temperatures from sowing to germination, °C
g-fAT	Sum of active temperatures from germination to flowering, °C
f-mAT	Sum of active temperatures from flowering to maturity, °C
s-mAT	Sum of active temperatures from sowing to maturity, °C
	**Total precipitation for the growing season**
s-gP	Total precipitation from sowing to germination, mm
g-fP	Total precipitation from germination to flowering, mm
f-mP	Total precipitation from flowering to maturity, mm
s-mP	Total precipitation from sowing to maturity, mm
g-mP	Total precipitation from germination to maturity, mm
	**Hydrothermal coefficient (HTC = P × 10/ΣAT) for the growing season**
s-gHTC	Hydrothermal coefficient from sowing to germination
g-fHTC	Hydrothermal coefficient from germination to flowering
f-mHTC	Hydrothermal coefficient from flowering to maturity
s-mHTC	Hydrothermal coefficient from sowing to maturity
	**Sum of active temperatures per growing season period**
may1dAT	Sum of active temperatures in the 1st decade of May, °C
may2dAT	Sum of active temperatures in the 2nd decade of May, °C
may3dAT	Sum of active temperatures in the 3rd decade of May, °C
mayAT	Sum of active temperatures in May, °C
jun1dAT	Sum of active temperatures in the 1st decade of June, °C
jun2dAT	Sum of active temperatures in the 2nd decade of June, °C
jun3dAT	Sum of active temperatures in the 3rd decade of June, °C
junAT	Sum of active temperatures in June, °C
jul1dAT	Sum of active temperatures in the 1st decade of July, °C
jul2dAT	Sum of active temperatures in the 2nd decade of July, °C
jul3dAT	Sum of active temperatures in the 3rd decade of July, °C
julAT	Sum of active temperatures in July, °C
aug1dAT	Sum of active temperatures in the 1st decade of August, °C
aug2dAT	Sum of active temperatures in the 2nd decade of August, °C
aug3dAT	Sum of active temperatures in the 3rd decade of August, °C
augAT	Sum of active temperatures in August, °C
may-augAT	Sum of active temperatures from May to August, °C
	**Sum of effective temperatures** (>20 °C) **per growing season period**
mayET	Sum of effective temperatures in May, °C
junET	Sum of effective temperatures in June, °C
julET	Sum of effective temperatures in July, °C
augET	Sum of effective temperatures in August, °C
may-augET	Sum of effective temperatures from May to August, °C
jun-julET	Sum of effective temperatures from June to July, °C
	**Total precipitation per growing season period**
may1dP	Total precipitation in the 1st decade of May, mm
may2dP	Total precipitation in the 2nd decade of May, mm
may3dP	Total precipitation in the 3rd decade of May, mm
mayP	Total precipitation in May, mm
jun1dP	Total precipitation in the 1st decade of June, mm
jun2dP	Total precipitation in the 2nd decade of June, mm
jun3dP	Total precipitation in the 3rd decade of June, mm
junP	Total precipitation in June, mm
jul1dP	Total precipitation in the 1st decade of July, mm
jul2dP	Total precipitation in the 2nd decade of July, mm
jul3dP	Total precipitation in the 3rd decade of July, mm
julP	Total precipitation in July, mm
aug1dP	Total precipitation in the 1st decade of August, mm
aug2dP	Total precipitation in the 2nd decade of August, mm
aug3dP	Total precipitation in the 3rd decade of August, mm
augP	Total precipitation in August, mm
may-augP	Total precipitation from May to August, mm
	**Hydrothermal coefficient**
mayHTC	Hydrothermal coefficient in May
junHTC	Hydrothermal coefficient in June
julHTC	Hydrothermal coefficient in July
augHTC	Hydrothermal coefficient in August
may-augHTC	Hydrothermal coefficient for the period from May to August

## Data Availability

All data are presented in the article.

## Supplementary Materials

The following supporting information can be downloaded at https://www.mdpi.com/article/10.3390/plants13060762/s1, Table S1: Temperature, precipitation and hydrothermal coefficient in 1987–2018; Table S2: Characteristics of fiber flax varieties Svetoch and Prizyv 81 by economically valuable traits, as well as by the sums of temperatures and precipitation during the growing seasons in 1987–2018; Table S3: Initial and z-transformed correlations between the economically valuable traits of flax; Table S4: Factor loading (Varimax raw) for 45 characters; Table S5: Factor scores of 7 factors for 30 years and factor loads for the years of evaluation, as well as the classification of the years of testing based on the results of grouping by factor loads using cluster analysis (K-means method); Table S6: Cluster analysis; Figure S1: Scree plot showing eigenvalues in response to the number of components for the estimated characters.
