# Supplementary material for: Influence of Weather Conditions in the Northwestern Russian Federation on Flax Fiber Characters According to the Results of a 30-Year Study
Source: Plants (Basel). 2024 Mar 7;13(6):762. doi: 10.3390/plants13060762 (PMC10975659; doi:10.3390/plants13060762)

Figure S1. Scree plot showing eigenvalues in response to the number of components for the estimated characters

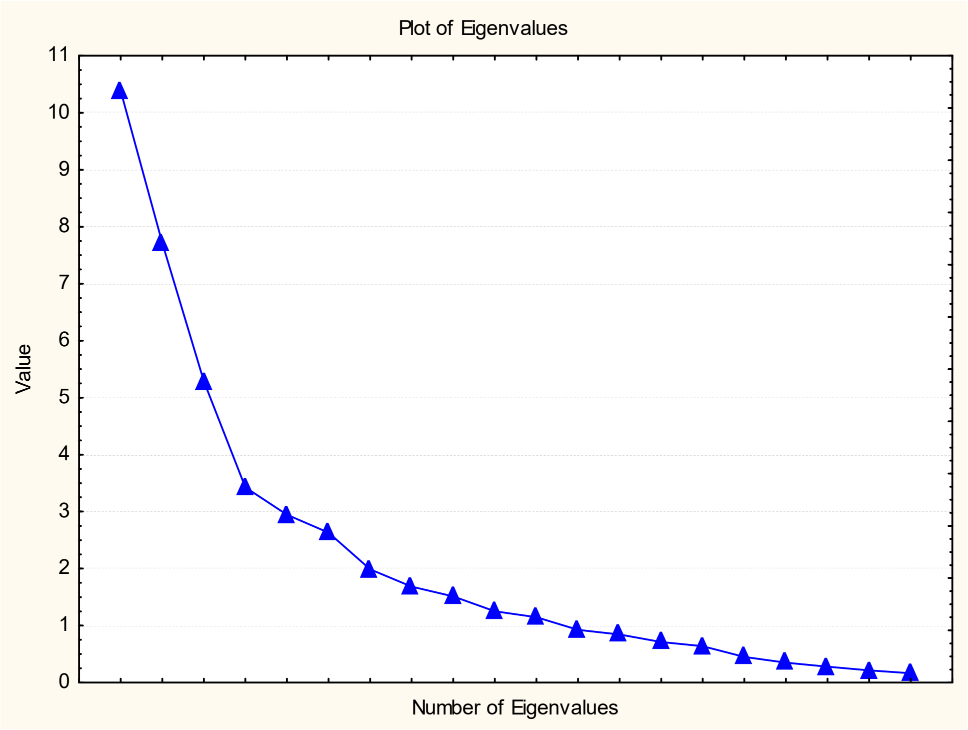

Supplement: Supplementary file 1 [file plants-13-00762-s001.zip › Figure S1.pdf]
