# Supplementary material for: Influence of Weather Conditions in the Northwestern Russian Federation on Flax Fiber Characters According to the Results of a 30-Year Study
Source: Plants (Basel). 2024 Mar 7;13(6):762. doi: 10.3390/plants13060762 (PMC10975659; doi:10.3390/plants13060762)
Supplement: Supplementary file 1 [file plants-13-00762-s001.zip › Supplement cont.pdf]

Table S1. Temperature, precipitation and hydrothermal coefficient in 1987-2018.
Table S2. Characteristics of fiber flax varieties Svetoch and Prizyv 81 by economically valuable characteristics, as well as by the sums of temperatures and precipitation during the growing seasons in 1987-2018
Table S3. Initial and z-transformed correlations between economically valuable flax characters
Figure S1. Scree plot showing eigenvalues in response to the number of components for the estimated characters
Table S4. Factor loading (Varimax raw) for 45 characters
Table S5. Factor scores of 7 factors for 30 years and factor loads for the years of evaluation, as well as the classification of the years of testing based on the results of grouping by factor loads using cluster analysis (method K-means)
Table S6. Analysis of Variance of Factors for Cluster analysis
