# Supplementary material for: Influence of Weather Conditions in the Northwestern Russian Federation on Flax Fiber Characters According to the Results of a 30-Year Study
Source: Plants (Basel). 2024 Mar 7;13(6):762. doi: 10.3390/plants13060762 (PMC10975659; doi:10.3390/plants13060762)
Supplement: Supplementary file 1 [file plants-13-00762-s001.zip › Table S4.pdf]

Table S4. Factor loading (Varimax raw) for 45 characters

**Characters**	Factor 1	Factor 2	Factor 3	Factor 4	Factor 5
may1dAT	-0,06	-0,07	0,12	0,24	0,20
may2dAT	0,49	-0,28	0,22	0,44	-0,15
may3dAT	0,22	0,01	0,37	0,52	0,28
mayAT	0,33	-0,16	0,38	0,63	0,20
jun1dAT	0,17	0,07	0,73	0,14	-0,27
jun2dAT	0,10	0,10	0,43	0,11	0,18
jun3dAT	0,21	0,04	0,66	0,00	0,25
junAT	0,23	0,10	0,89	0,13	0,04
jul1dAT	0,46	0,00	0,38	0,07	-0,27
jul2dAT	0,81	0,06	-0,08	0,03	0,29
jul3dAT	0,82	-0,12	-0,10	0,23	-0,01
julAT	0,85	0,01	0,14	0,14	-0,10
aug1dAT	0,55	0,10	-0,14	0,57	-0,23
aug2dAT	-0,07	0,10	0,06	0,82	0,04
aug3dAT	0,05	0,27	0,24	0,43	0,02
augAT	0,24	0,23	0,08	0,86	-0,08
may-augAT	0,59	0,02	0,51	0,59	0,05
may1dP	0,15	0,03	-0,27	-0,13	-0,59
may2dP	-0,13	0,04	0,56	0,22	-0,34
may3dP	0,09	0,10	-0,11	0,24	-0,76
mayP	0,05	0,09	0,11	0,21	-0,94
jun1dP	0,07	-0,30	-0,26	-0,31	-0,13
jun2dP	0,30	-0,73	-0,33	0,06	0,05
jun3dP	0,12	-0,62	-0,18	-0,15	-0,28
junP	0,21	-0,76	-0,34	-0,20	-0,19
jul1dP	-0,40	-0,33	-0,10	0,17	0,15
jul2dP	0,47	-0,08	0,14	-0,04	0,36
jul3dP	-0,25	-0,47	0,44	0,13	0,18
julP	-0,30	-0,53	0,25	0,18	0,30
aug1dP	-0,27	-0,68	0,42	-0,22	0,07
aug2dP	0,28	-0,45	0,08	-0,19	0,32
aug3dP	-0,02	-0,65	-0,21	0,29	0,33
augP	-0,02	-0,85	0,09	0,00	0,34
may-augP	-0,03	-0,99	0,04	0,04	0,02
mayHTC	-0,16	0,16	-0,14	-0,23	-0,87
junHTC	0,17	-0,73	-0,45	-0,20	-0,18
julHTC	-0,52	-0,52	0,17	0,16	0,31
augHTC	-0,07	-0,84	0,09	-0,17	0,29
may-augHTC	-0,22	-0,94	-0,12	-0,15	-0,01
mayET	0,41	0,03	0,05	0,44	-0,06
junET	0,24	0,11	0,85	-0,02	0,18
julET	0,89	0,02	0,05	0,06	-0,04
augET	0,27	0,16	-0,06	0,75	-0,29
may-augET	0,82	0,12	0,37	0,35	-0,06
jun-julET	0,83	0,08	0,47	0,04	0,06
Eigenvalues	7,22	7,79	5,43	4,97	4,34
Proportion of variance	0,16	0,17	0,12	0,11	0,10
Square root from proportion variance	0,40	0,42	0,35	0,33	0,31
