# Supplementary material for: Influence of Weather Conditions in the Northwestern Russian Federation on Flax Fiber Characters According to the Results of a 30-Year Study
Source: Plants (Basel). 2024 Mar 7;13(6):762. doi: 10.3390/plants13060762 (PMC10975659; doi:10.3390/plants13060762)
Supplement: Supplementary file 1 [file plants-13-00762-s001.zip › Table S5.pdf]

**Table S5**. Factor scores of 7 factors for 30 years and factor loads for the years of evaluation, as well as the classification of the years of testing based on the results of grouping by factor loads using the cluster analysis (K-means method)

| | Factor scores | | | | | K-means classification | |
|---|---|---|---|---|---|---|---|
| Year | Factor 1 | Factor 2 | Factor 3 | Factor 4 | Factor 5 | Number of cluster | Distance |
| 1987 | -0,95 | -1,85 | 0,14 | -1,85 | -1,84 | 2 | 1,09 |
| 1988 | 1,19 | -0,93 | 1,04 | -1,21 | 1,51 | 6 | 0,60 |
| 1989 | -1,16 | -0,99 | 2,22 | -0,26 | 0,43 | 7 | 0,71 |
| 1990 | -1,49 | 1,10 | -0,60 | -0,36 | -0,10 | 4 | 0,65 |
| 1991 | -0,04 | 0,42 | -0,49 | -0,76 | -0,84 | 2 | 0,42 |
| 1992 | -0,27 | 1,42 | -0,34 | -0,18 | 1,26 | 4 | 0,40 |
| 1994 | 0,56 | 0,10 | -1,19 | -1,54 | -0,39 | 2 | 0,64 |
| 1995 | -0,83 | 0,80 | 1,30 | -0,39 | -0,77 | 3 | 0,76 |
| 1996 | -1,88 | 0,63 | -0,13 | 0,52 | -1,62 | 3 | 0,58 |
| 1997 | 0,17 | 1,34 | -0,12 | -0,32 | -0,38 | 4 | 0,47 |
| 1998 | -0,44 | -0,93 | -0,09 | -1,15 | -0,21 | 2 | 0,41 |
| 1999 | 0,84 | 1,16 | 1,26 | -1,82 | 0,64 | 6 | 0,60 |
| 2000 | -0,85 | 0,20 | -0,11 | -0,18 | 0,61 | 4 | 0,49 |
| 2001 | 0,66 | 0,23 | -0,47 | -0,95 | -0,17 | 2 | 0,49 |
| 2002 | 0,06 | 1,15 | 0,01 | 0,64 | 0,64 | 4 | 0,33 |
| 2004 | -0,54 | -0,69 | -0,87 | 0,33 | -0,65 | 2 | 0,57 |
| 2005 | 0,18 | -0,49 | -0,03 | -0,09 | -0,71 | 2 | 0,33 |
| 2006 | -0,05 | 0,96 | 0,44 | 0,50 | -0,05 | 4 | 0,45 |
| 2007 | -0,38 | 0,22 | -0,83 | 1,69 | -1,11 | 3 | 0,66 |
| 2008 | -1,36 | -0,28 | -0,88 | -0,24 | 1,40 | 5 | 0,54 |
| 2009 | 0,24 | -0,50 | -1,53 | -0,31 | 1,42 | 5 | 0,63 |
| 2010 | 2,25 | -1,05 | -1,25 | 0,05 | -1,53 | 1 | 0,47 |
| 2011 | 1,26 | 0,09 | 0,99 | -0,05 | -0,06 | 6 | 0,56 |
| 2012 | 0,55 | -0,61 | -0,10 | 0,50 | 0,13 | 2 | 0,68 |
| 2013 | 0,63 | -0,33 | 2,63 | 1,33 | -0,82 | 7 | 0,76 |
| 2014 | 2,03 | 0,02 | -0,57 | 1,60 | -0,88 | 1 | 0,47 |
| 2015 | -0,57 | 0,94 | 0,46 | 1,78 | -0,18 | 3 | 0,56 |
| 2016 | 0,70 | -0,89 | 0,92 | 0,95 | 1,53 | 7 | 0,75 |
| 2017 | -1,16 | -2,60 | -0,78 | 1,54 | 1,46 | 5 | 0,88 |
| 2018 | 0,63 | 1,36 | -1,05 | 0,24 | 1,27 | 4 | 0,66 |
